# Computational metabolomics reveals flavonoid architectures underlying species-specific chemical identity in medicinal *Plectranthus*

**DOI:** 10.1007/s11306-026-02546-x

**Published:** 2026-10-25

**Authors:** Akhona Myoli, Ntakadzeni E. Madala, Farhahna Allie, Justin J. J. van der Hooft, Fidele Tugizimana

**Affiliations:** 1 Research Centre for Plant Metabolomics, Department of Biochemistry, University of Johannesburg, Auckland Park, Johannesburg, South Africa; 2 Department of Biochemistry and Microbiology, University of Venda, Thohoyandou, 0950 South Africa; 3 Bioinformatics Group, Wageningen University & Research, Wageningen, 6708 PB the Netherlands

**Keywords:** Computational metabolomics, GNPS, MS2LDA 2.0, MS2Query, Natural products, Flavonoids, *Plectranthus*

## Abstract

**Introduction**: Plant metabolomes encode biochemical information underlying species diversity, ecological adaptation, and pharmacological potential. Within the genus *Plectranthus*, diterpenoids are widely regarded as both the primary bioactive constituents and species-specific chemical signatures; however, the extent to which other bioactive metabolite classes contribute to species-specific chemical signatures remains poorly understood.

**Objective**: To characterize and compare the leaf metabolomes of *Plectranthus hadiensis* and *Plectranthus ambiguus*, two medicinally important species used in South African ethnopharmacology.

Methods: Methanolic leaf extracts of P. hadiensis and P. ambiguus were analysed usinguntargeted LC–MS/MS platform. The spectral data were mined and annotated using feature-based molecular networking followed by comparative metabolomics and GNPS library matching, MS2Query; and MS2LDA 2.0-enabled structure and substructure-level annotations.

**Results**: The findings revealed extensive metabolic diversity beyond diterpenoids. While the prenol lipid chemical class, consisting of diterpenoids, was confirmed as a major discriminatory chemical class, flavonoids emerged as key drivers of species-level chemical differentiation. Distinct structural patterns were observed, with glycosylated flavones predominating in *P. hadiensis*, whereas methylated flavones and glycosylated flavonols were enriched in *P. ambiguus*. These compositional differences reflect underlying variation in flavonoid biosynthetic modifications, suggesting divergent metabolic regulation between species.

**Conclusions**: Computational metabolomics revealed that flavonoid structural diversity constitutes a previously underappreciated layer of chemical specialization in *Plectranthus* plants and contributes substantially to species-specific chemical identity beyond the emphasized diterpenoids. By moving beyond targeted phytochemical screening, this work establishes a scalable framework for resolving species-specific metabolic architectures in medicinal plants. These findings have implications for chemotaxonomy, natural product discovery, and the rational exploration of plant-derived bioactives, contributing to a deeper understanding of the molecular basis of ethnomedicinal efficacy.

**Supplementary Information:**

The online version contains supplementary material available at https://doi.org/10.1007/s11306-026-02546-x.

## Introduction

Throughout history, plants have played and continue to play a key role in the discovery and development of pharmaceutical drugs and multifunctional cosmeceuticals (Nicolai et al., 2020; Chaachouay & Zidane, 2024). Among these plants, the genus *Plectranthus* has been recognized for its bioactive compounds, which have garnered interest in its pharmacological potential (Guo, 2017; Newman & Cragg, 2020). Also known as spurflowers, the genus comes from the family Lamiaceae and consists of approximately 300 species that are distributed through the tropical and warm regions of the world including Africa, India and Australia (Arumugam et al., 2016; Marquesl et al., 2012). The worldwide distribution of *Plectranthus* species translates to an assortment of ethnomedicinal uses that vary culturally from region to region. In South Africa, *P. ambiguus* (known as iBoza elincane in isiZulu) and *P. hadiensis* (known as iLozane in isiXhosa) grow freely in the southern KwaZulu-Natal (KZN) and the northern parts of the Eastern Cape (EC) provinces. Phenotypically, the small leaves of *P. ambiguus* ((Bolus) Codd) are easily distinguishable from the large leaves of *P. hadiensis* ((Forssk.) Schweinf. ex Spreng.) – this is one of the morphological differences between the two plant species (Van Wyk and Smith 2001; Van Jaarsveld,  2006; Hao et al., 2022).

Furthermore, *P. ambiguus* and *P. hadiensis* are among the traditionally utilized *Plectranthus* species in South Africa and the broader southern African region. Their ethnomedicinal applications show considerable overlap, with *Plectranthus* species being used for the treatment of various ailments, including respiratory conditions, skin infections, fever and inflammation (Rice et al., 2011; Matias et al., 2019). At the same time, differences in the reported traditional applications of individual species suggest that their medicinal uses may not be entirely equivalent: *P. ambiguus* has been particularly associated with the treatment of respiratory ailments, whereas *P. hadiensis* has also been reported for digestive and gastrointestinal conditions (Lukhoba et al., 2006; Rice et al., 2011). This longstanding ethnomedicinal relevance, together with the similarities and distinctions in their traditional uses, provides a rationale for comparatively investigating the chemical composition of *P. ambiguus* and *P. hadiensis*. Previous studies have identified that *Plectranthus* species, including *P. ambiguus* and *P. hadiensis* are particularly rich in specialized metabolites such as diterpenoids (Abdel-Mogib et al., 2002). For example, modified abietane-type diterpenoids are frequently highlighted as the major bioactive constituents contributing to the pharmacological properties of these plants. Additionally, recently, other classes of compounds such as phenolic acids and flavonoids have also been associated with their biological activity (Śliwiński et al., 2020; Lambrechts & Lall, 2021; Barbosa et al., 2023).

However, prior phytochemical studies have largely focused on the classical screening methods targeting these well-known abietane diterpenoids (Kubínová et al., 2014; Śliwiński et al., 2020; Sitarek et al., 2020; Ntungwe et al., 2021). While these studies have provided valuable insights, they offer only a partial view of the overall chemical repertoire of these species. Based on the available literature, as far as we know, no comprehensive metabolomic comparison has been conducted between *P. ambiguus* and *P. hadiensis*. Such knowledge gaps on the metabolomic landscape of these species restricts our understanding of their phytochemical complexity and potentially overlooks other important bioactive compounds. Moreover, the exclusive focus on diterpenoids constrains our ability to identify shared or species-specific metabolic fingerprints that may explain differences or similarities in their traditional uses and biological activities.

Thus, reported herein is a comprehensive metabolomics-based approach to investigate and compare the complex metabolite profiles of *P. hadiensis* and *P. ambiguus*. This untargeted, computational metabolomics work integrates feature finding with MZmine 4, comparative metabolomics and annotation tools available within Feature-based Molecular Networking (FBMN), as well as MS2Query and MS2LDA 2.0 (Heuckeroth et al., 2024, Nothias et al., 2020, de Jonge et al., 2023; Van der Hooft et al., 2016) to visualize and uncover hidden layers of chemical diversity that may underpin species-level metabolic differentiation. As such, the work enables a holistic exploration of the chemical diversity and chemical signatures of these two traditionally important South African medicinal plants. This comparative analysis contributes to bridging indigenous knowledge with modern analytical science, expanding the current understanding of *Plectranthus* phytochemistry beyond classical diterpenoid profiles by focusing on the understudied flavonoid chemical class in the genus. Thus, through the application of an integrated computational metabolomics pipeline, this study, provides new insights into the chemical differentiation and potential pharmacological significance of *P. ambiguus* and *P. hadiensis*.

## Experimental procedure

### Metabolite extraction and sample preparation

*Plectranthus ambiguus* ((Bolus) Codd) and *Plectranthus hadiensis* ((Forssk.) Schweinf. ex Spreng.) plants (60 days old) were purchased from Random Harvest Nursery in Krugersdorp, South Africa. The taxonomic identity of the plants was confirmed through the nursery’s record-keeping and accession systems. At Randon Harvest, the plants were grown in 4 kg bags under natural outdoor conditions during a South African summer season (14 November 2023 to 14 January 2024). During this period, average daytime temperatures ranged from 25 to 29 °C, while nighttime temperatures ranged from 14 to 17 °C. The leaves of the plants were harvested, air-dried, and crushed into fine powder. For metabolite extraction, 200 mg of plant material was accurately measured and dissolved in 10 mL of 80% methanol (Chemlab, UK). The samples were then placed in a digital rotisserie tube rotator (Thermo Fisher, Johannesburg, South Africa) at 70 rpm and spun overnight. The crude extracts were centrifuged afterwards at 1330 rpm at 4 °C using a benchtop fixed-angle centrifuge (Thermo Fisher, Johannesburg, South Africa). The supernatants, after centrifugation, were filtered using 0.22 μm nylon filters into glass vials and stored at 4 °C until further analysis. For this study, five independent biological replicates were used.

### Data acquisition – LC-MS/MS analysis of crude methanolic leaf extracts

Samples (methanol extracts) were analyzed on a liquid chromatography–quadrupole time-of-flight mass spectrometry (LC-qToF-MS) system (LCMS-9030, Shimadzu Corporation, Kyoto, Japan). Chromatographic separation was achieved by injecting 3 µL sample onto a Shim-pack C18 column (100 mm × 2.1 mm, 2.7 μm) (Shimadzu Corporation, Kyoto, Japan) maintained at 55 °C. The reverse-phase chromatographic separation was performed using a binary mobile phase and a gradient elution program. The solvent system consisted of solvent A (0.1% formic acid (HPLC grade, Merck, Darmstadt, Germany) in Milli-Q water) and solvent B (consisting of methanol (HPLC grade, Romil SpS, Cambridge, UK) with 0.1% formic acid), with a flow rate of 0.4 mL/min. The gradient elution was performed as follows, B referring to organic composition (i.e., solvent B): 5% B for 3 min, 5–40% B over 3–5 min, 40–95% B over 5–12 min, then 95% B for from 12 min to 18 min. The gradient was changed back to initial 5% B at 18 min and kept to 20 min. The column was re-equilibrated for 3 min. The column effluents from chromatographic separation were further analysed with the high-definition mass spectrometer, equipped with electrospray ionization (ESI). Following method optimization, mass spectral data were acquired in negative ionization mode based on optimal ion efficiency. The mass spectrometer was operated under the following conditions: 4.0 kV interface voltage, with interface temperature of 300 °C; 3 L/min flow rate for nebulization and dry gas; DL temperature of 250 °C, and 400 °C for heat block; detector was operated at 1.8 kV voltage. For monitoring the accuracy of acquired mass-to-charge ratio (*m/z*), sodium iodide (NaI) was used as a calibration solution. Both full scan (MS^1^) and tandem MS/MS (MS^2^) spectral data were acquired over an *m/z* range of 100–1200 Da. MS/MS experiments were conducted using a data-dependent acquisition (DDA) method with an intensity threshold of 5000 counts. Ion fragmentation was achieved via Collision-Induced Dissociation (CID) method using argon as a collision gas at a collision energy of 30 eV with a spread of 5 eV.

### Peak detection and alignment

The acquired raw data files in the mzML format (ESI negative centroid data) were processed using MZmine 4.8.30. All preprocessing parameters were determined through an initial inspection of the overall quality of the dataset by assessing the mzML files. After the inspection and optimization of the data preprocessing steps on MZmine, the following parameters were used: for MS1 mass detection, the factor of the lowest signal parameter was selected with the corresponding noise factor set at 8.0, and for MS2, it was set at 1.1. Thereafter, the parameters for the chromatogram builder were set as follows: the minimum consecutive scans were set to 5, with the minimum intensity for consecutive scans and minimum absolute height set at 6.0E3 and 7.0E3, respectively. To resolve the detected peak features, the chromatographic threshold was set at 85% with the minimum search range retention time (RT) set at 0.04. Sample alignment was then performed with the weight for *m/z* and RT set at 3 and 1, respectively, to generate an aligned feature list. Gap filling of the aligned feature list was then performed with the intensity threshold set at 20%. The resulting gap-filled and aligned feature list (peak area matrix) was composed of 3531 features (detected peaks). The full set of MZmine processing parameters are provided as a batch file in Zenodo (Doi: https://doi.org/10.5281/zenodo.20112661).

### Statistical analysis and identification of discriminant markers

The feature quantification table, exported from MZmine, was then used to generate ecological diversity metrics and multivariate chemometric models. Using each feature as a unique entry, ecological diversity metrics (Shannon and Simpson indices) were calculated to determine metabolite diversity (equations in Sect. 1 of the Supplementary file) following normalization of the feature quantification table (peak area matrix) using the total sum scaling. The Shannon and Simpson diversity indices were computed using the vegan package in R. To compute the multivariate chemometric models, the feature quantification table was imported into soft independent modelling of class analogy (SIMCA) version 18.0 software (Sartorius, South Africa). Principal Component Analysis (PCA) and Orthogonal Partial Least Square-Discriminant Analysis (OPLS-DA) models were computed for data exploration, group classification, and discriminant analysis.

### Computational strategies for metabolite annotation

Post MZmine data processing, the GNPS export files, both the spectral .mgf file and feature quantification table, were exported into the newly released GNPS2 ecosystem (Wang et al., 2016) and FBMN was then computed. The parameters used for computing FBMN for the spectral dataset included precursor ion mass tolerance of 0.25 Da with fragment ion mass tolerance of 0.15 Da. For spectral similarity, a cosine score cut-off was set at 0.65, with a minimum of 5 matched fragment ions. The default GNPS2 spectral libraries (all reference spectra in the LC library irrespective of the ionization mode) were used for metabolite annotation. To visualize and analyze the computed network, the Cytoscape network visualization software (version 3.8.2) (Shannon et al., 2003; Smoot et al., 2011) was used. All putative node annotations and some unmatched/unidentified nodes, visualized in Cytoscape, were verified using their empirical formulae calculated from accurate mass and fragmentation patterns. In addition to the manual inspection of annotations, PubChem (https://pubchem.ncbi.nlm.nih.gov/) was also used to verify the exact spectral library matches obtained from GNPS, MS2Query and MS2LDA by comparing the fragmentation patterns of each annotation to its corresponding PubChem entry.

In addition to annotations sourced from GNPS spectral library matching, MS2Query (version 1.5.4) was also carried out for analogue and exact metabolite. MS2Query is a tool that enables high-throughput MS2 library searching, supporting the simultaneous detection of structural analogues and exact spectral matches. The tool provides reliable predictions for both analogues and exact library matches using MS2 information (de Jonge et al., 2023). The same mass spectral file (.mgf file) used for FBMN was used as an input file for MS2Query. Subsequently, the MS2Query run was executed via the command line in Python 3.9, following the script provided on its official GitHub page (https://github.com/iomega/ms2query) and using the negative ESI mode MS2Query library obtained at (https://zenodo.org/record/7104184). The results were then overlaid on the FBMN using the feature IDs as keys.

To further enhance and increase confidence in the putative metabolite annotations, substructure (Mass2Motifs) annotations were carried out using the MS2LDA 2.0 (MS2 Latent Dirichlet allocation version 2.0) (Van der Hooft et al., 2016). The revised scalable workflow of MS2LDA 2.0 enables substructure exploration using MS2 information with new innovative features such as the Spec2Vec-powered automated Mass2Motif Annotation Guidance (MAG) system and queryable and matchable MotifDB MotifSets, while simultaneously leveraging Python-based metabolomics frameworks such as matchms to handle the mass spectrometry data (Huber et al., 2021; Ortega et al., 2025). For the ESI negative dataset acquired in this study, MS2LDA 2.0 was implemented using a Python script provided on its official Github page (https://github.com/vdhooftcompmet/MS2LDA/blob/main/README_CONDA.md) and the model for negative ionization mode was downloaded at https://zenodo.org/records/17521464. The MS2LDA settings included setting the number of discovered motifs to 399 with the number of iterations set at 1000. For the downstream analysis, the results were then uploaded, visualized and further analyzed using the MS2LDAViz 2.0 application (https://ms2lda.org/viz/) where only the negative Motifset LDBs, namely LDB_NEG_MotifDB_01, LDB_NEG_MotifDB_02 and Motifset Rhamnaceae Plant MotifDB were selected as reference Motif Data Bases (MotifDBs). Annotated Mass2Motifs that matched with a similarity score ≥ 0.3 to experimental Mass2Motifs were considered relevant to follow-up on to check if their annotations could be transferred. Within Mass2Motifs, mass spectra with both a probability and overlap score ≥ 0.2 were considered associated to the Mass2Motif and accounting to its degree.

Following the compilation of verified putative annotations from GNPS2 and MS2Query, metabolite class annotations were automatically assigned using ClassyFire (http://classyfire.wishartlab.com/), a chemical class ontology and classification tool integrated into MS2Query and GNPS2 where neighbouring nodes were assigned class annotations based on the ClassyFire-classified structures identified through library matching or analogue search. Data visualization was also done using circlize version 0.4.17 (Gu et al., 2014) in R. All metabolite annotations were carried out at level 2 and 3 of the Metabolomics Standards Initiative (MSI) (Sumner et al., 2007; Schymanski et al., 2014), and all validated annotations (manual and computational) are listed in the Supplementary Table 1.

## Results and discussion

**Fig. 1:**
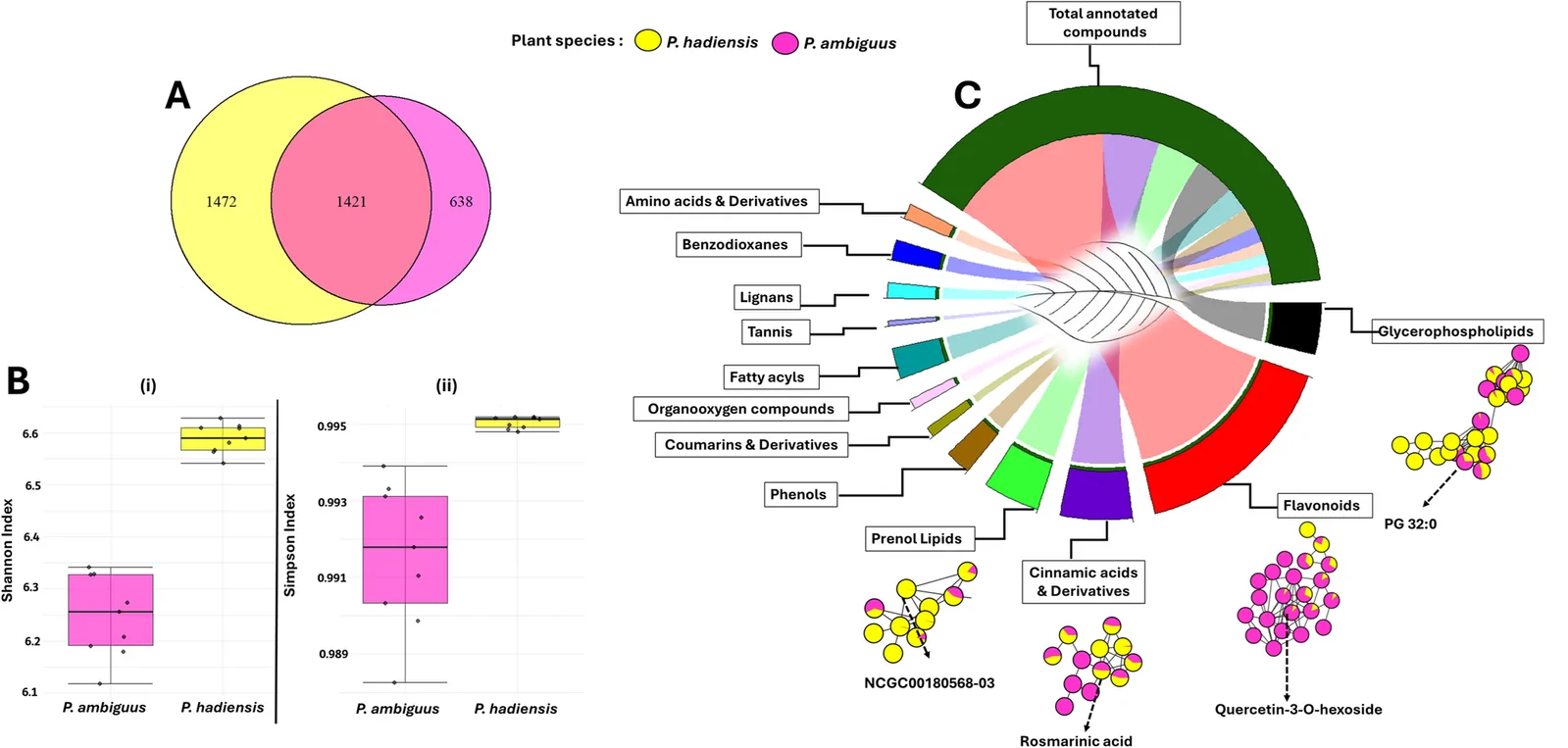
Chemical complexity and diversity of metabolite profiles in P. hadiensis and P. ambiguus. **A** Overview of the detected metabolite features, highlighting shared and unique features between both species**B** Diversity indices reflecting metabolite diversity: **i** Shannon index and **ii** Simpson index. **C** Total number of annotations belonging to the glycerophospholipids, cinnamic acids, prenol lipids and flavonoids chemical classes as highlighted by each molecular family in the FBMN. The highlighted molecular families are comprised of metabolites shown as interconnected nodes. Some of the metabolites in each molecular family were identified through GNPS spectral library matching MS2query, MS2LDA 2.0 and manual annotation. The pie charts represent the relative distribution of each metabolite across *P. hadiensis* and *P. ambiguus*

Methanolic leaf extracts of *P. hadiensis* and *P. ambiguus* (five biological replicates per plant species) were analyzed using an LC-qToF-MS system. Visual inspection of the base peak chromatograms acquired in negative ESI mode revealed distinct differences in peak presence and intensity, which highlighted the differential metabolite profiles between *P. hadiensis* and *P. ambiguus* (Figure S1). Following data pre-processing, a comparative metabolomics analysis of the generated spectral data revealed 1421 shared metabolite features out of a total of 3531 between *P. hadiensis* and *P. ambiguus*, indicating a conserved metabolic inventory between the two plant species. Nonetheless, *P. hadiensis* exhibited 1472 unique metabolite features, suggesting a higher metabolite diversity and a higher degree of divergence from the metabolite profile of *P. ambiguus*, which in turn displayed 638 unique features (Fig. 1A). To further explore the degree of divergence in the metabolite profiles of the two species, metabolite diversity was assessed using ecological metrics, namely the Shannon and Simpson diversity indices.

Although these indices are traditionally applied in community ecology studies to quantify biodiversity, they are also useful in measuring chemodiversity across different plant species. In this study, metabolite diversity analysis provides a statistical measure of chemical variability within the metabolomes of the studied plant species. Thus, in this context, using the untargeted metabolomics dataset, individual metabolite features are used as input and treated as unique entries (i.e., “biological species”) with their relative abundances corresponding to species abundance (Peters et al., 2021; Mansoldo et al.,^2022^).

Therefore, the Shannon index as an output, is highly influenced by the presence of rare metabolites and reflects both the richness and evenness of metabolite distribution. In contrast, the Simpson index places greater emphasis on the dominance of highly abundant metabolites (Morris et al., 2014; Santini et al., 2017; Chaura et al., 2026). In this study, Fig. 1B(i) shows the Shannon index values where *P. ambiguus* exhibited a lower value, suggesting less metabolite diversity defined by the reduced richness and evenness of metabolite distribution. Conversely, *P. hadiensis* showed the highest diversity, indicating a broader and more evenly distributed metabolite profile. Similarly, the Simpson index values as shown by Fig. 1B(ii), further pointed to *P. hadiensis* as having a more diverse metabolome, which could be used in explaining the species’ environmental adaptability and bioactivity potential.

The significance of these two diversity indices was evaluated using the Wilcoxon rank-sum tests, where both the Shannon and the Simpson indices yielded a p-value of 4.11 x $\:{10}^{-5}$, respectively, demonstrating a statistically significant difference in metabolic complexity and diversity between the two species. Following the initial exploratory metabolite diversity analysis, which revealed a heterogeneous chemical complexity between two plant species, the FBMN approach enabled the visualization of the chemical space of the species’ leaf extracts by organizing features into molecular families based on MS/MS spectral similarity. FBMN revealed structural relationships among the detected metabolites (shared and unique features), where each metabolite is represented as a node (Fig. S2). The nodes in the molecular network were annotated using GNPS spectral library matching, complemented with additional in-silico annotation tools such as MS2Query and MS2LDA 2.0. Notably, 43% of the total verified annotations were correctly identified as exact matches (*m/z* difference ≤ 0.003) by MS2Query with prediction scores ≥ 0.5. The reliable in-silico compound predictions not only expanded the annotations but also increased confidence in the annotations obtained through GNPS spectral library matching and manual annotations.

These structural annotations uncovered a broad spectrum of metabolite classes. These discovered chemical classes (based on the compound classifications enabled by ClassyFire in MS2Query and GNPS2) included cinnamic acids, flavonoids, prenol lipids, benzodioxanes, glycerophospholipids, coumarins, and tannins (Fig. 1C). While several metabolite features were common to both species, distinct clusters of nodes were uniquely associated with either *P. hadiensis* or *P. ambiguus*, suggesting species-specific chemical signatures as illustrated earlier by Fig. 1A. Although a substantial number (1421 metabolite features) of metabolites are shared between the two species, their species-specific chemical signatures, observed in some of the molecular families, reflect underlying metabolic variation and may explain, in part, their differing traditional uses and pharmacological profiles. For example, *P. hadiensis* has been reported to treat digestive conditions, whereas *P. ambiguus* is mostly reported for treating respiratory infections (Lukhoba et al., 2006; Rice et al., 2011). However, network-based visualization alone does not quantify the statistical significance or relative contribution of individual features to species differentiation. To further interrogate and validate the observed species-specific chemical signatures, comparative metabolomics analysis with unsupervised and supervised chemometric models was conducted. This chemometric approach complements FBMN by providing a quantitative assessment of the observed differential metabolic profiles across the two species, facilitating the identification of key discriminating features that define the species-specific metabolic differences.

### Chemometric analysis identifies flavonoids as key drivers of species-specific chemical differentiation

**Fig. 2:**
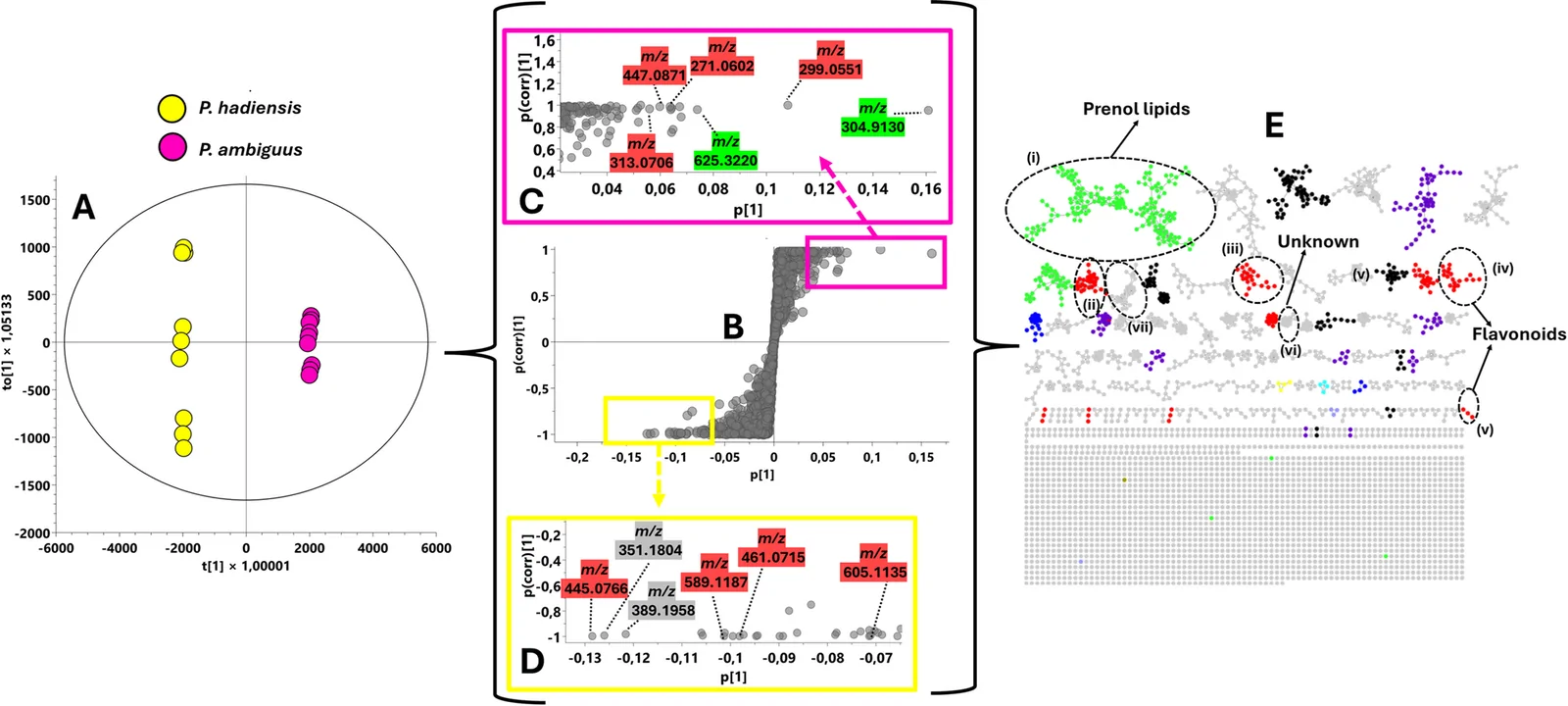
Comparative metabolomics differentiate the metabolomes of Plectranthus species. OPLS-DA scores plot (**A**) of *P. hadiensis* and *P. ambiguus* methanolic leaf extracts showing species-specific sample grouping. **B–D** S-plot generated from the OPLS-DA model, highlighting the discriminating metabolite features (box on the upper right (**C**) and bottom left (**D**)) responsible for the observed species-specific sample grouping. **E** Some of the statistically significant discriminating metabolite features highlighted in (**C** and **D**) were mapped on the FBMN of P. hadiensis and P. ambiguus and were revealed to belong to the prenol lipid (**i**) and flavonoid (**ii**,** iii**,** iv** and **v**) chemical classes, with some mapped on an unknown MF (**vi** and **vii**)

Both unsupervised and supervised statistical approaches, PCA and OPLS-DA were computed as described in Sect. 2.4. The PCA scores plot of the Pareto-scaled dataset (Fig. S3) showed distinct sample grouping where *P. ambiguus* samples were observed to group away from *P. hadiesnsis* samples, highlighting the underlying metabolite differences between the two plants. This observation was further explored by applying a supervised approach to determine the discriminating metabolite features between the two species. The OPLS-DA method enables the identification of metabolite features that are significantly contributing to the separation of two well-defined sample groups (Trygg et al., 2007; Tugizimana et al., 2013). The generated OPLS-DA model, as a binary classifier, separating multivariate relationships into predictive (species differentiation) and orthogonal (unrelated to species differentiation) variation (Fig. 2A), was validated by assessing the robustness, predictive ability, reliability and significance of the models. The computed and validated OPLS-DA model (p ˂ 0.05) (Fig. 2A) was a perfect classifier and statistically reliable, with very good predictive capability: no signs of possible overfitting, as indicated by cross-validation; and none of the permutated models (Fig. S3B) performed better than the original model in separating classes, *P. hadiensis* and *P. ambiguus*.

Some of these validations included a 7-fold cross-validation, with R^2^ and Q^2^ metrics, the analysis of variance testing of cross-validated predictive residuals (CV-ANOVA, *p*-value ˂ 0.05 as a cut-off), the receiver operator characteristic (ROC) curves and response permutation tests (Fig. S3B-C) (Eriksson et al., 2014; Tugizimana et al., 2016; Westerhuis et al., 2008). Furthermore, the selection of discriminating metabolite features (*P. hadiensis* vs. *P. ambiguus*) was carried out by evaluating the OPLS-DA loading S-plot (Fig. 2B–D). To avoid overinterpretation of the model and reduce variable selection bias, only features that were statistically significant in contributing to species separation were retained. Hence, variables that combined both high covariation and correlation (as examined on S-plot) were statistically relevant as potential discriminant metabolite features (Tugizimana et al., 2013; Wiklund et al., 2008). However, it is important to note that the S-plot is susceptible to data matrix changes due to correlation sensitivity and dependency on data structure.

Therefore, in this study, additional tests and tools such as the variable importance in projection (VIP) plots, jackknife confidence intervals (used to estimate standard errors in a nonparametric way as an estimate of bias) (Fig. S3D) were used to further confirm the statistical significance and discriminability of the S-plot-derived potential discriminant markers (Galindo-Prieto et al., 2015; Gika et al., 2014; Tugizimana et al., 2016). Furthermore, from the descriptive statistics, some of the top relevant markers discriminating *P. ambiguus* from *P. hadiensis* are highlighted by Fig. 2C and include *m/z* 313.0706, *m/z* 447.0871, *m/z* 271.0602, *m/z* 625.3220, *m/z* 299.0551 and *m/z* 304.9130. On the other hand, *m/z* 389.1958, *m/z* 589.1187, *m/z* 461.0715, *m/z* 351.1804 and *m/z* 445.0766 (Fig. 2D) were the most relevant markers responsible for discriminating *P. hadiensis* from *P. ambiguus*. The statistically significant markers for both plant species were observed to belong to the prenol lipid and flavonoid chemical classes with some features (351.1804 and *m/z* 389.1958) mapped on MFs that had no chemical class identity on the FBMN (Fig. 2E). Since the two species are predominantly known for their prenol lipid (e.g., diterpenoids) content, the flavonoid chemical class was further investigated for its contribution to the species-specific patterns observed in the chemical landscape of *P. ambiguus* and *P.hadiensis*.

### Flavonoid structural diversification shapes species-specific chemical identity

The combination of chemometric models and mass spectral annotation tools (namely FBMN, MS2Query, and MS2LDA 2.0) pointed to flavonoids as one of the major chemical classes responsible for the metabolite diversity as depicted by the differences in the distinct metabolite profiles of *P. ambiguus* and *P. hadiesis* (Fig. 2). The discriminating features were further evaluated by considering VIP scores, where only metabolite features with VIP scores > 2 were retained as discriminating markers driving the metabolite differences. The criterion for the VIP value was set at > 2 (instead of the commonly used criterion of ≥ 1) to reduce and narrow down a smaller subset of metabolites that are the most relevant features in discriminating the two species. For instance, from the metabolite features that are positively correlated to *P. hadiensis*, *m/z* 445.0766 on the S-plot with a high VIP score of 7.45, was putatively identified through FBMN as baicalin. After baicalin, sorbifolin (*m/z* 299.0551) was observed to have the second highest VIP score of 6.62 among the metabolite features that are positively correlated to *P. ambiguus*, indicating the flavonoid content as a key discriminatory chemical class between *P. hadiesnis* and *P. ambiguus*.

**Fig. 3:**
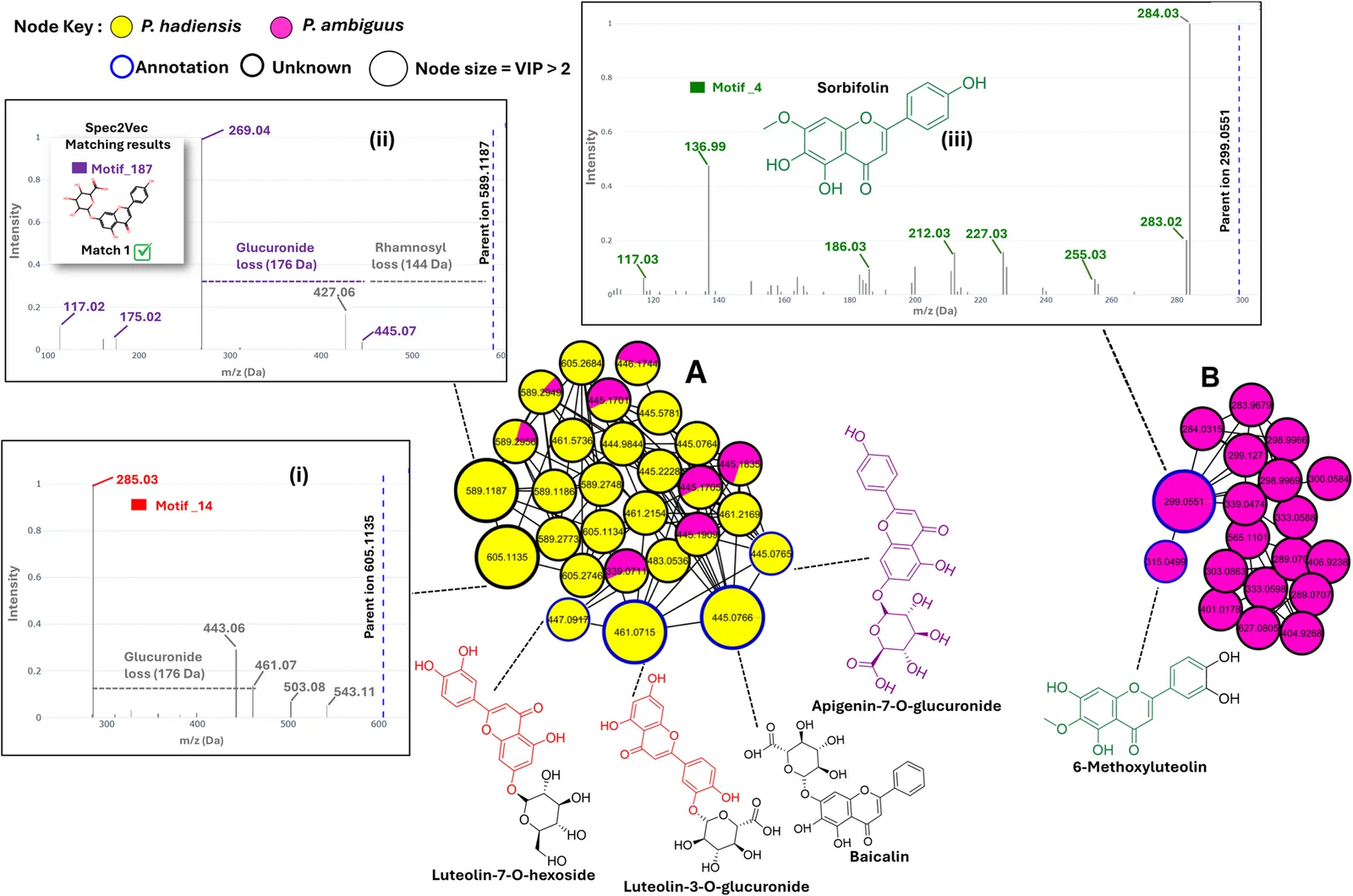
MS2LDA 2.0 substructure discovery on the computed FBMN.** A** An MF of glycosylated flavones with the discovered Mass2Motifs, including (**i**) motif_14 (Luteolin related) and **ii** motif_187 (Apigenin-7-O-glucuronide related) annotated through Spec2Vec matching by Mass2Motif Annotation Guidance. **B** An MF of methylated flavones with **iii** motif_4 (Sorbifolin related). The node keys placed on the top left describe both **A** and **B** molecular families

When evaluating the baicalin molecular family (MF), we observed that additional discriminant markers (VIP score > 2) highly abundant in *P. hadiensis* were also present in the same MF. These markers included luteolin-3-O-glucuronide, unknown *m/z* 605.1135 and *m/z* 589.1187 (Fig. 3A). To further investigate the unknown discriminant markers in the baicalin MF, MS2LDA 2.0 was applied to assess the structural architecture and relationship between the known and unknown metabolites in the cluster. In the MS2LDA analysis, a total of 399 Mass2Motifs were detected across the dataset, with 18 Mass2Motifs detected in the baicalin MF (Fig. S4A). From the 18 main motifs in the MF, motif_14 and motif_187 were found in more than one node (Fig. S4A). When using MS2LDA and assessing the fragmentation spectra of *m/z* 605.1135, the structure of which remains unidentified, it was observed that *m/z* 605.1135 shared a common fragment (motif_14 = 285.03 Da) with luteolin-3-O-glucuronide and luteolin-3-O-hexoside (VIP score < 2) (Fig. 3A(i)).

Notably, in all three connected and structurally related parent ions, motif_14 (285.03 Da) as a shared substructure was found to be associated with luteolin. This was supported by the presence of a luteolin diagnostic fragment of 285.03 Da in the fragmentation pattern of *m/z* 605.1135, luteolin-3-O-glucuronide, and luteolin-3-O-hexoside, respectively. In luteolin-3-O-glucuronide, motif_14 further fragments to produce low-intensity luteolin diagnostic fragments of *m/z* 175.03 and *m/z* 133.02 (Fabre et al., 2001), reducing the probability of motif_14 being any other isomeric flavonoid aglycone; however, further validation experiments are required to fully verify the aglycone identity. Moreover, a glucuronide sugar moiety (neutral loss of 176 Da) specifically indicated that *m/z* 605.1135 is an analogue of luteolin-3-O-glucuronide (Fig. 3A(i)). The neighbouring node, unknown *m/z* 589.1187, was shown to be comprised of motif_187 (445 Da). Leveraging on the new Mass2Motif Annotation Guidance (MAG) in MS2LDA 2.0 that uses Spec2Vec (Huber et al., 2021) to find potential matches for parent ions in a large mass spectral library, a metabolite feature whose *m/z* value equals that of motif_187 was recommended to be apigenin-7-O-glucuronide, an annotation that was a spectral hit in GNPS2 and MS2query, respectively. Within the dataset, motif_187 was found to be common in 68 different metabolite features eluting at different times. The pseudospectra (optimized fragmentation peaks) of motif_187 reflected apigenin structural similarities as confirmed by the apigenin diagnostic fragments of *m/z* 269.04 and *m/z* 117.02 with glycosylation shown by the glucuronide sugar moiety (Fig. 3A(ii)).

Thus *m/z* 589.1187 was postulated to be a glycosylated analogue of apigenin-7-O-glucuronide with an additional rhamnosyl sugar moiety. The presence and abundance of these glycosylated flavonoids in *P. hadiensis* may have functional significance in the context of medicinal plant applications. For instance, glycosylation plays a vital role in the medicinal properties of flavonoids as it increases the reactivity, solubility and stability of the corresponding bioactive aglycones, thus improving bioavailability and bioactivity of flavonoids (Alseekh et al., 2020). Notwithstanding the predominance of glycosylated flavones in *P. hadiensis*, simple hydroxylated flavone aglycones, namely apigenin (*m/z* 269.0445) and luteolin (*m/z* 285.0393), were also identified as highly abundant discriminating features in *P. hadiensis* (Fig. S5). Conversely, methylated flavones were observed to be highly abundant discriminating features in *P. ambiguus* and included a molecular family containing sorbifolin (VIP score > 2) (Fig. 3B).

**Fig. 4:**
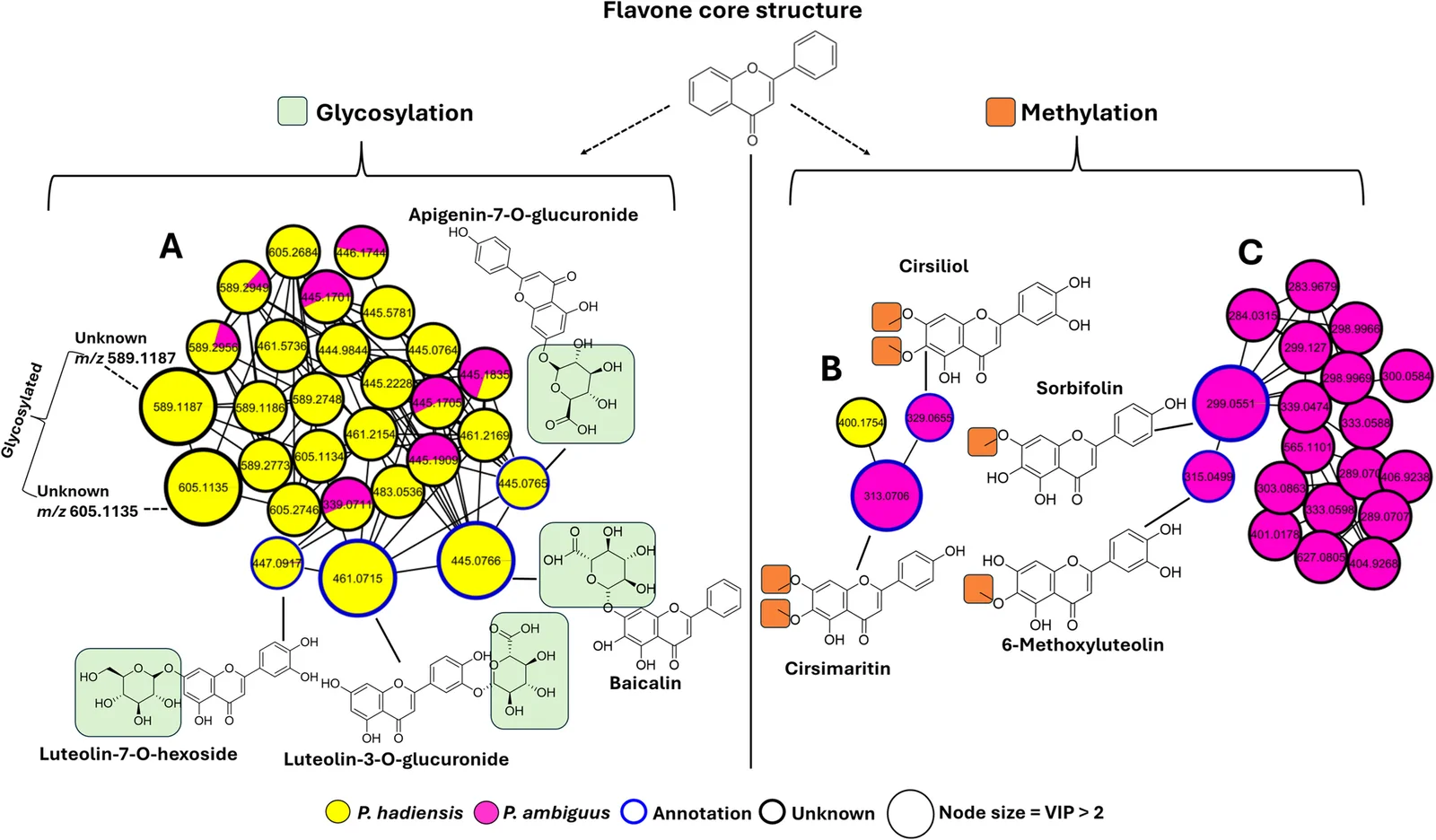
Flavone molecular families (MFs) from the generated FBMN (Fig. S2) with glycosylation and methylation modifications in *P. hadiensis* and *P. ambiguus*. **A** An MF of glycosylated flavones consisting of luteolin 3-O-glucuronide (*m/z* 605.1135), baicalin (*m/z* 445.0766), *m/z* 589.1187 and *m/z* 605.1135 as discriminating metabolite features (VIP score > 2) while **B, C** are MFs of methylated flavones consisting of cirsimaritin (m/z 313.0706) sorbifolin (*m/z* 299.0551) as discriminating metabolite features (VIP score > 2). Glycosylation and methylation are highlighted with the green and orange boxes, respectively. The pie charts indicate the relative abundance of metabolite features across the two plant species

Amongst the 16 Mass2Motifs detected in the sorbifolin MF, motif_4 was the most common substructure (Fig. S4B). The Spec2Vec matching of MAG suggested the chemical annotation of motif_4 to be 5,7-dihydroxy-3-(4-hydroxyphenyl)-6-methoxychromen-4-one, an isomer of sorbifolin. The probability and overlap score for motif_4 detected in the mass spectra of sorbifolin (*m/z* 299.0551) was observed to be 0.326 and 0.559 respectively. Guided by this and the generated pseudospectra of motif_4, comprised of fragments 284.03 Da, 283.04 Da, 255 Da, 227.03 Da, 212.03 Da, 186.03 Da, 136.99 Da and 117.03Da, we annotated motif_4 as being related to a sorbifolin (sub)structure. This was further supported by the pseudospectra of motif_4, which corresponded and overlapped with the fragmentation pattern of sorbifolin as deposited in the PubChem database (CID:3084390). Therefore, this further increased the confidence in the GNPS-generated annotation of the neighbouring node (*m/z* 315.0499) as 6-methoxyluteolin, since both metabolites are structural analogues of each other with varying hydroxylation and methylation patterns. Although methylated flavonoids generally occur in lower levels than their glycosylated counterparts in plants, it has been suggested that differences in polarity or the accumulation of methylated hydrophobic flavones in medicinal plants, as seen in *P. ambiguus*, could point to specific biological roles (Berim & Gang, 2016). Interestingly, when assessing the glycosylated and methylated discriminant flavonoids in Fig. 3A, B, it was observed that they share a flavone backbone (C6-C3-C6 form) with similar hydroxylation modifications (Fig. 4).

However, the overall chemical architecture of the discriminant flavonoids (VIP score > 2) is distinguished by glycosylation and methylation modifications, respectively, a trend that also carries over to the neighbouring nodes in each MF (Fig. 4A and B). These flavone structural differences are reflected at the species level by the prevalence of flavone glycosylation in *P. hadiensis* (Fig. 4A), whereas flavone methylation was more prominent in *P. ambiguus* (Fig. 4B, C). Therefore, both glycosylation and methylation modifications can be postulated as possible chemical signatures that differentiate the leaf tissues of the two plant species.

**Fig. 5:**
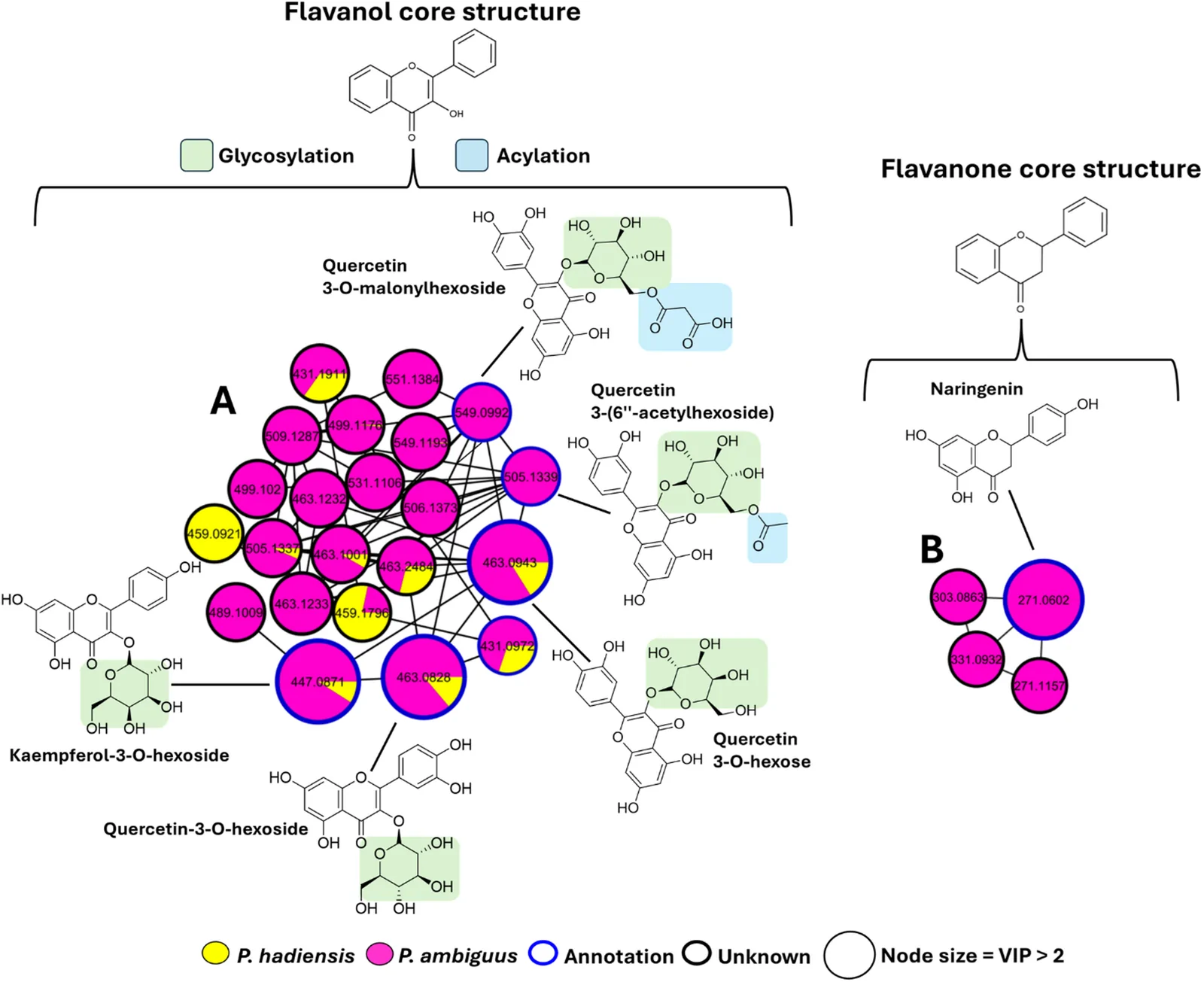
FBMN showing **A** flavanols and **B** flavonones *P. hadiensis* and *P. ambiguus*, with the sugar moieties highlighted by the green boxes in addition to the acetyl and malonyl conjugates highlighted by blue boxes, respectively. **A** Glycosylated flavonols consisting of kaempferol-3-O-hexoside (*m/z* 447.0671) and quercetin-3-O-hexoside (*m/z* 463.0828) as discriminating metabolite features. **B** A flavonone MF consisting of naringenin (*m/z* 271.0602) as a discriminating metabolite feature. The pie charts on both **A** and **B** indicate the relative abundance of flavonols and flavonones in *P. ambiguus* and *P. hadiensis*

Upon further investigations into the flavonoid chemical class, it was observed that discriminant flavonols were also one of the flavonoid subclasses, differentiating the leaf metabolite profiles of the two species. Notably, flavonols (Fig. 5A) and flavanones (Fig. 5B) were observed to be predominantly present in *P. ambiguus* than in *P. hadiensis*. The core structure of flavonols differs from that of the flavones due to the presence of a hydroxyl group on the C-ring at the C-3 position (Todorova et al., 2013; Stachelska et al., 2025; Przybylski et al., 2025). As mentioned earlier, the chemical features and biological activities of flavonoids are influenced by the nature of the substituents attached to the basic structures. Free hydroxyl groups are more reactive than glycosylated and methylated hydroxyl groups, and this is vital for various flavonoid biological activities, such as antioxidant activities.

Thus, structure and activity studies have shown that the presence of the hydroxyl group on the C-ring of flavonols, which often undergoes modifications, is one of the substituents that is frequently associated with the antioxidant activities of flavonoids (Halbwirth, 2010; Verdan et al., 2011). Stachelska et al., 2025; Przybylski et al., 2025). Consequently, the C-ring hydroxyl group of flavanols can undergo glycosylation where a sugar moiety is attached to C-3. In this study, glycosylation on the two flavanol aglycones resulted in the formation of two quercetin-3-O-hexoside metabolites (potentially a glucoside and a galactoside) and kaempferol-3-O-hexoside, exact metabolite matches as identified through both FBMN and MS2query (Fig. 5A).

**Fig. 6:**
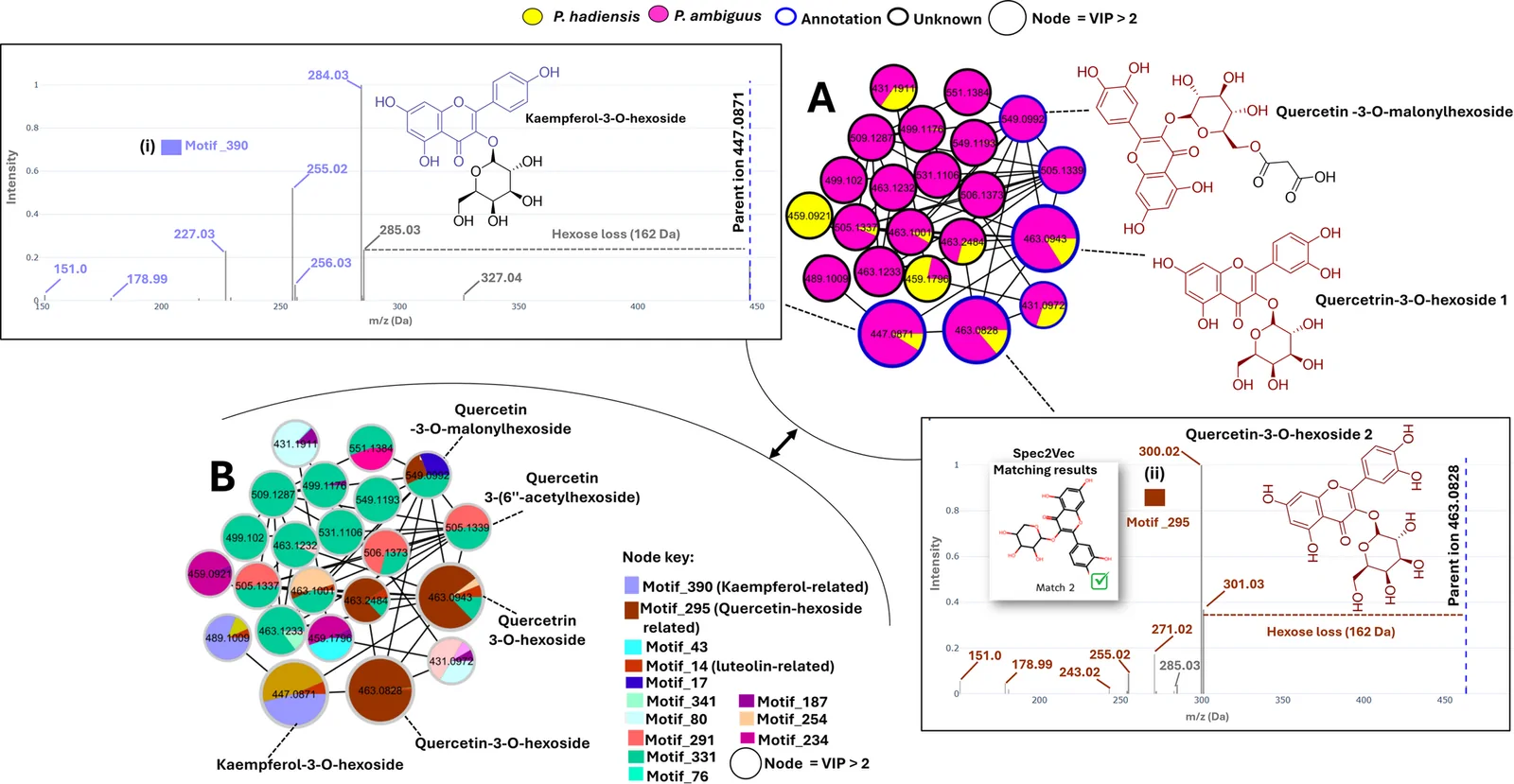
MS2LDA 2.0 substructure discovery on the computed FBMN. **A** An MF of glycosylated flavanols with duplicate MF **B** showing 16 discovered Mass2Motifs, including) **i** motif_390 (Kaempferol related) and **ii** motif_295 (quercetin-3-O-hexoside related). The node key at the top describes MF (**A**), where the pie charts indicate the relative abundance of metabolite features across the two plants species while the pie charts in duplicate MF **B** indicate the discovered Mass2Motifs listed at the bottom

Both annotations were further confirmed by the Mass2Motifs discovered through MS2LDA 2.0 as shown by the duplicate MF highlighting all discovered Mass2Motifs (Fig. 6B). From the 14 substructures discovered in the MF, one of the Mass2Motifs in kaempferol-3-O-hexoside, was motif_390, initially detected as an unknown (Fig. 6B). This Mass2Motif (motif_390), was later identified as a kaempferol-related substructure based on its pseudospectra, which included kaempferol diagnostic fragments such as 284.03 Da, 225.02 Da and 151.0 Da (Fig. 6A(i)) (Fabre et al., 2001).

The metabolite features annotated with quercetin-3-O-hexoside were associated with motif_295, and the MAG Spec2Vec matching functionality returned the quercetin-3-O-hexoside annotation as a Mass2Motif annotation of motif_295 (Fig. 6A(ii)), which increased confidence in the GNPS spectral library and MS2Query-generated annotations. As discussed, glycosylation increases the hydrophilicity of flavonoid aglycones, minimizes toxic side effects, and enhances the specific targeting ability of flavonoids (Hu et al., 2025). Amongst the glycosyl moieties, an additional attachment is seen in the form of acylation (highlighted in blue on Fig. 5A) and is illustrated by the putative annotation of quercetin-3-O-malonylhexoside and quercetin-3-(6”-acetylhexoside) (Fig. 5A). The acylation of the sugar moieties is crucial for enhancing molecular stability, improving water solubility, and increasing resistance to enzymatic degradation of natural products (Urakawa et al., 2024). Various plant species carry out flavonoid malonylation, a type of acylation reaction, in which malonyltransferases transfer a malonyl group from malonyl-CoA with regioselectivity to a hydroxyl group of a flavonoid sugar residue, a reaction common in anthocyanins and flavanols (Kogawa et al., 2007; Kachlicki et al., 2008). Additionally, acetyltransferases facilitate the transfer of acetyl groups from various donors to flavonoid glycosyl moieties (Wang et al., 2022). In addition to influencing the physico-chemical properties of flavonoids, acetylation of sugar residues has been identified as contributing to the cytoprotective effects and anti-inflammatory effects in in vivo studies (Urakawa et al., 2024).

In addition to the predominance of modified flavanols, it was observed that within the flavanone subclass, naringenin (VIP score = 3.89) was also one of the significant discriminating features in *P. ambiguus* plants (Fig. 5B and Fig. S5A(i)). In the naringenin MF, unknown metabolite features (*m/z* 303.0863 and *m/z* 331.0932) were observed to be modified derivatives of naringenin, based on their mass spectral fragmentation patterns that suggested methylation (Fig. S6A(ii) and S6A(iii)). The duplicate MF highlighted 9 Mass2Motifs (Fig. S6B) where the parent ion masses, namely *m/z* 331.0932 (Fig. S6A (ii)) and *m/z* 303.0863 (Figure S6A(iii)) both were shown to have a naringenin-related substructure (motif_258), confirmed by the presence of the naringenin diagnostic fragment of 271 Da and 119 Da. Both parent ions showed an initial neutral loss of 32 Da, suggesting the loss of a methoxy group.

This observation was further supported by the MS2query analogue annotation of *m/z* 331.0932 as naringenin 5,7-dimethyl ether (precursor analogue *m/z* 299 in negative ESI), suggesting possible methylation on the unknown metabolite features. Naringenin is well known for its biological activities, which range from antioxidant to anti-diabetic properties (Shilpa et al., 2023; Pansare et al., 2025). However, naringenin as an aglycone is known to have poor water solubility, which affects its overall bioavailability and bioactivity (Arafah et al., 2020; Uçar & Göktaş, 2023). The presence of methylated derivatives (*m/z* 303.0863 and 331.0932) of naringenin could potentially enhance the bioavailability of naringenin and thus positively benefit the overall medicinal properties of *P. ambiguus*.

The elucidation and comparative study of the metabolite profiles of *P. hadiensis* and *P. ambiguus* has highlighted flavonoids and their respective chemical modifications as species-specific chemical signatures that differentiate the two medicinal plant species. This type of chemo-characterization, driven by the integration of computational metabolomics tools, holds potential in understanding the environmental adaptation strategies and ethnopharmacological uses of these two species. Considering the lack of comprehensive data on the metabolite composition *P. ambiguus* and *P. hadiensis* metabolomes, the information generated in this study lays the foundation for generating insights into finding key metabolites that contribute to each species’ unique medicinal properties beyond their well-known diterpenoid content.

## Conclusion

An in-depth exploration of the metabolite composition of medicinally important *P. hadiensis* and *P. ambiguus* plants was achieved using computational metabolomics strategies. Metabolite annotation facilitated by the integration of mass spectral annotation tools such as feature-based molecular networking, MS2Query, and MS2LDA 2.0 revealed a diverse range of chemical classes that span the metabolic atlas of both plant species. The combination of comparative chemometric models and mass spectral annotation tools pointed to the flavonoid chemical class as one of the key chemical classes that may underpin the species-specific metabolite profiles of *P. hadiensis* and *P. ambiguus* leaf extracts. The findings from this study have provided new insights into the contribution of the differential flavonoid chemical architecture in the chemotaxonomic classification of *Plectranthus* species, where *P. hadiensis* was observed to be rich in glycosylated flavones, while *P. ambiguus* was characterized by the abundance of methylated flavones and glycosylated flavanols. Such observation brings to the forefront the need to explore the ecological relevance of metabolite diversification and its impact on the unique ethnomedicinal applications and biological activities elicited by each species. However, it is important to note that these observations may be influenced by plant-growing environmental conditions and/or unique metabolic biosynthetic pathways. Moreover, additional studies are required to evaluate the biological activities of each plant species in relation to the enriched plant-specific flavonoid subclasses. Nonetheless, the current study highlights the potential of *P. hadiensis* and *P. ambiguus* plants as treasure troves of natural products with therapeutic relevance, promoting further exploration of the two plant species under various environmental and experimental conditions, including complementary functional studies to confirm the ecological and biological relevance of both plant species.

## Supplementary Information

Below is the link to the electronic supplementary material.

Supplementary Material 1

## Data Availability

The mzmine 4.8.30 batch file, including all data preprocessing settings, together with the MS2LDA analysis config file specifying the analysis settings and the results file (.json) are all made available at Zenodo (Doi: 10.5281/zenodo.20112661). The spectral data presented in this study is made available on the supplementary files as a GNPS job link. The molecular networking files (feature table and mgf files) and raw (mzML) files can be found in MassIVE (MSV000101781).
