# Supplementary material for: Computational metabolomics reveals flavonoid architectures underlying species-specific chemical identity in medicinal Plectranthus
Source: Metabolomics. 2026 Oct 25;22(6):173. doi: 10.1007/s11306-026-02546-x (PMC13696593; doi:10.1007/s11306-026-02546-x)
Supplement: Supplementary file 1 [file 11306_2026_2546_MOESM1_ESM.pdf]

Supplementary Materials

Computational metabolomics reveals flavonoid architectures underlying species-specific chemical identity in medicinal *Plectranthus*

1. Equations for ecological matrices used for metabolite diversity analysis. 1
    1.1 Shannon index equation: 1
    1.2 Simpson index equation: 1
2. Base peak chromatograms 1
2. GNPS Feature-based molecular networking..2
3. Supplementary Figures 2-6 3

1. Equations for ecological matrices used for metabolite diversity analysis.

1.1 Shannon index equation:

$H = -\sum_{i=1}^{s} pi\, In(pi)$ where $In(pi)$ is the natural logarithm of the relative abundance (proportion) of metabolite i.

1.2 Simpson index equation:

$D = \sum_{i=1}^{s} (\frac{n_i}{N})^2$, where n_i is the number of individual metabolites in the species and N is the total of all metabolites.

2. Base peak chromatograms

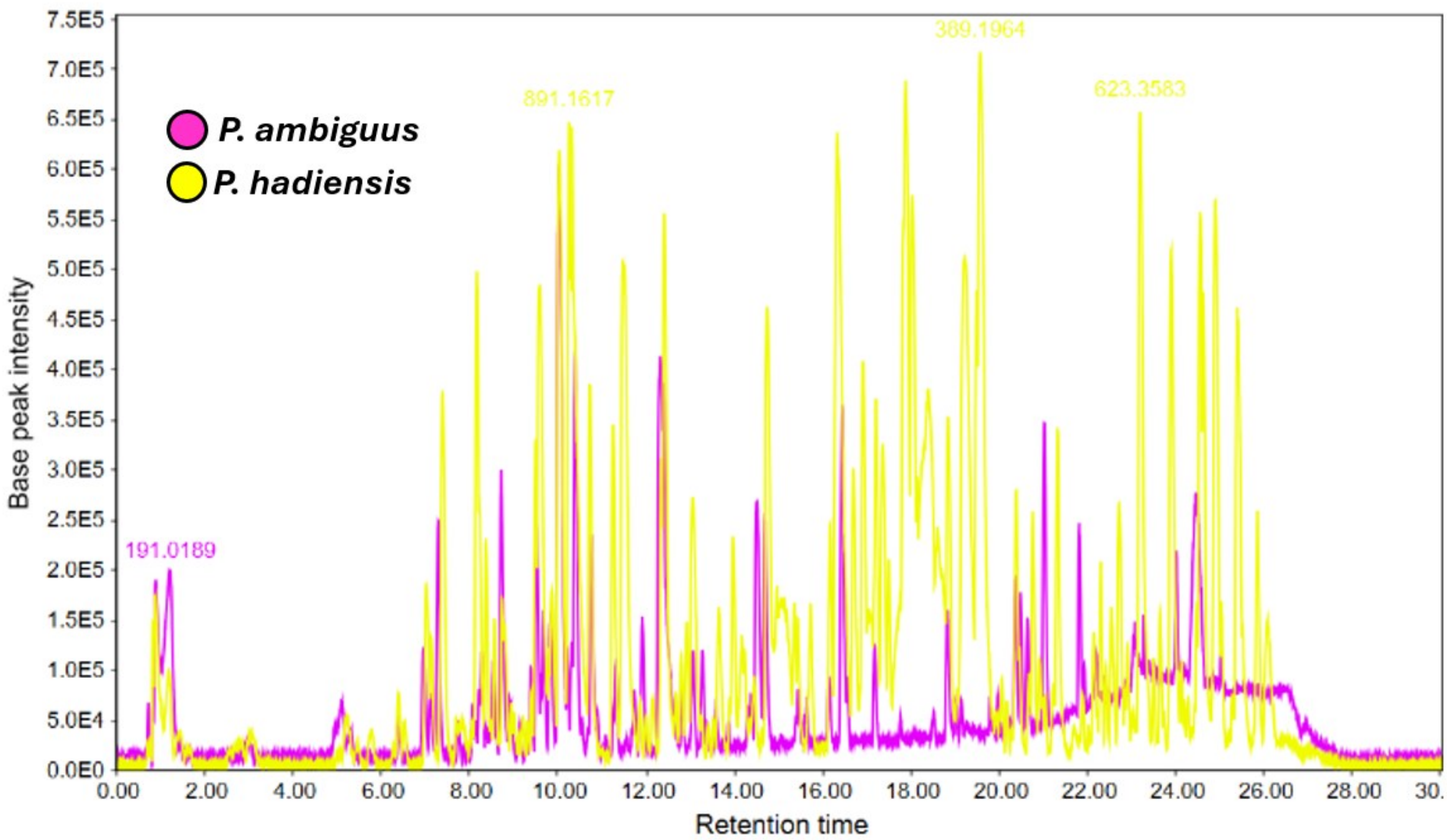

Figure S1: Representative base peak chromatograms acquired in negative ESI mode showing the differential metabolite profiles of *P.ambiguus* and *P.hadiensis*.

2. GNPS Feature-based molecular networking

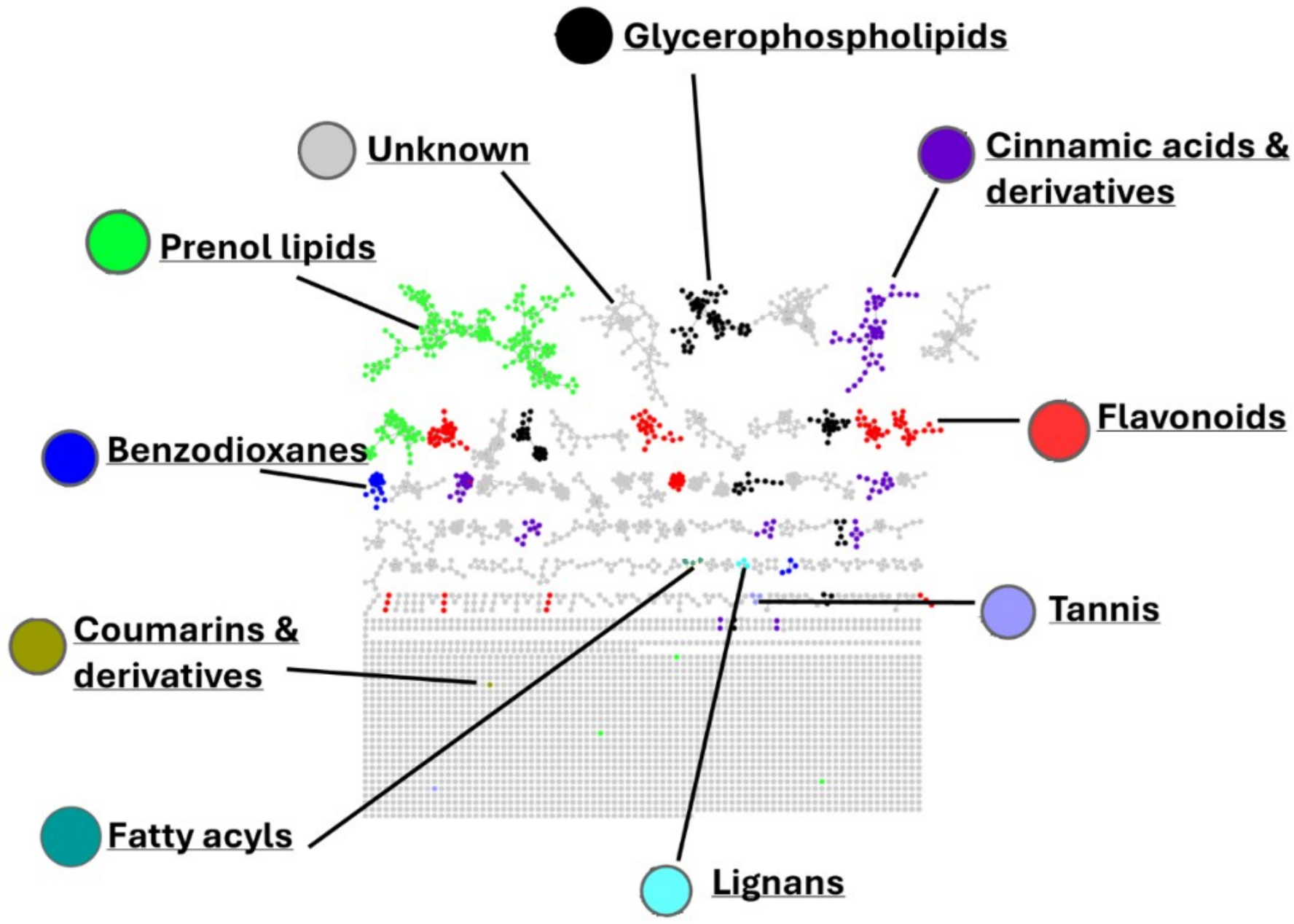

Figure S2: FBMN of *P.ambiguus* and *P.hadiensis* showing some of the detected chemical compound classes based on the ClassyFire ontology class annotations in MS2Query and GNPS2.

GNPS job link: https://gnps2.org/status?task=6091d0274f0642fb8abf5033d70ab4b6

3. Supplementary Figures 2-6

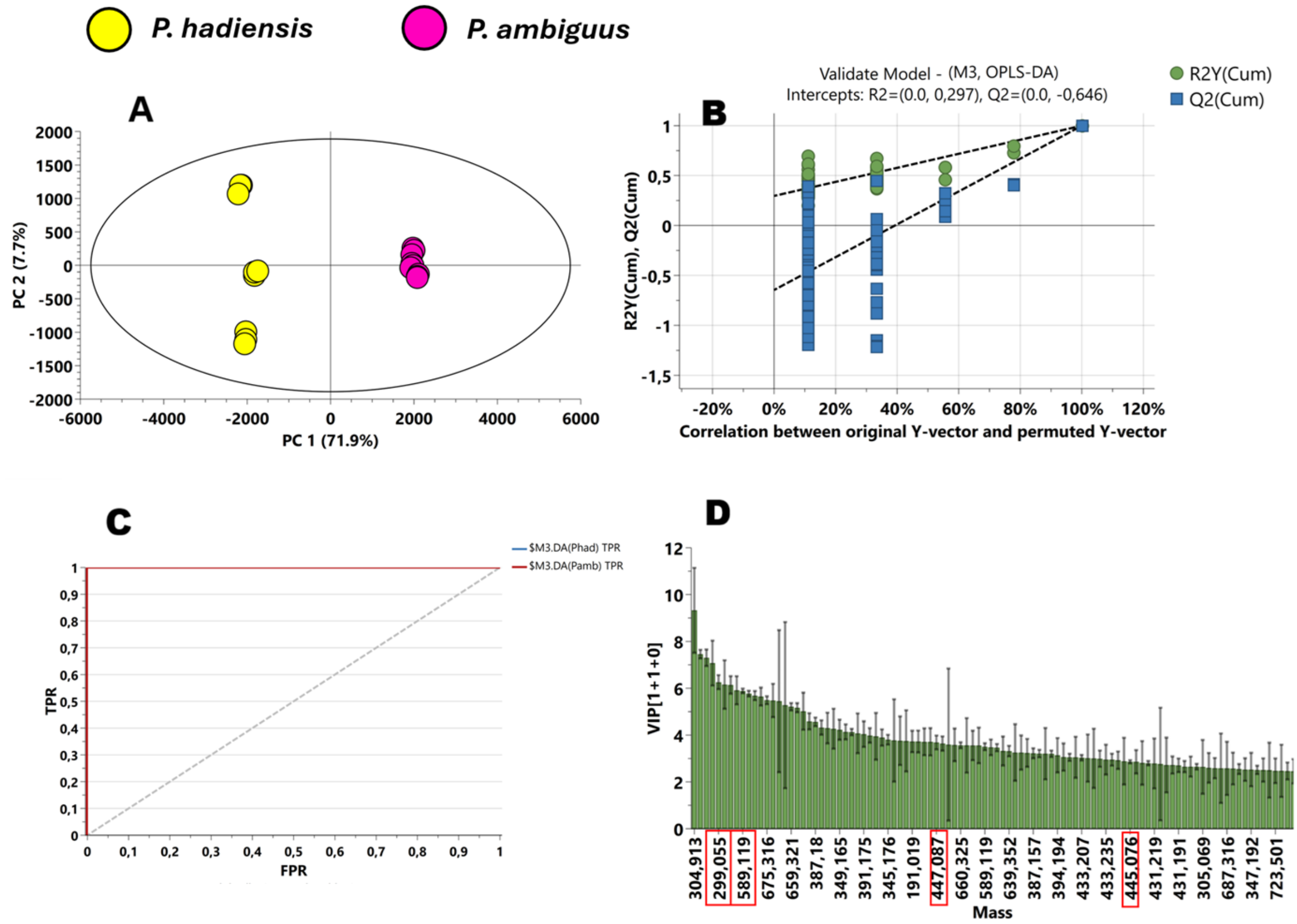

Figure S3: A n-components PCA model (**A**), explaining 96.6% of the total variation (R2), with a predictive variation of 89.1% (Q2). (**B-D**) OPLS-DA model validation where (**B**) = Permutation test plot (with n=100), (**C**) = Receiver Operator Characteristic (ROC) plot for the OPLS-DA model separating *P. hadiensis* vs *P. ambiguus* samples (1+1+0 components, R^2= 0.777 and Q^2 = 0.999, CV-ANOVA p-value = 2.8 X 10^{-20}) and (**D**) = variable importance for the projection (VIP) plot pointing to the important metabolite features, including annotated flavonoids (red boxes) contributing to species separation on the OPLS-DA model.

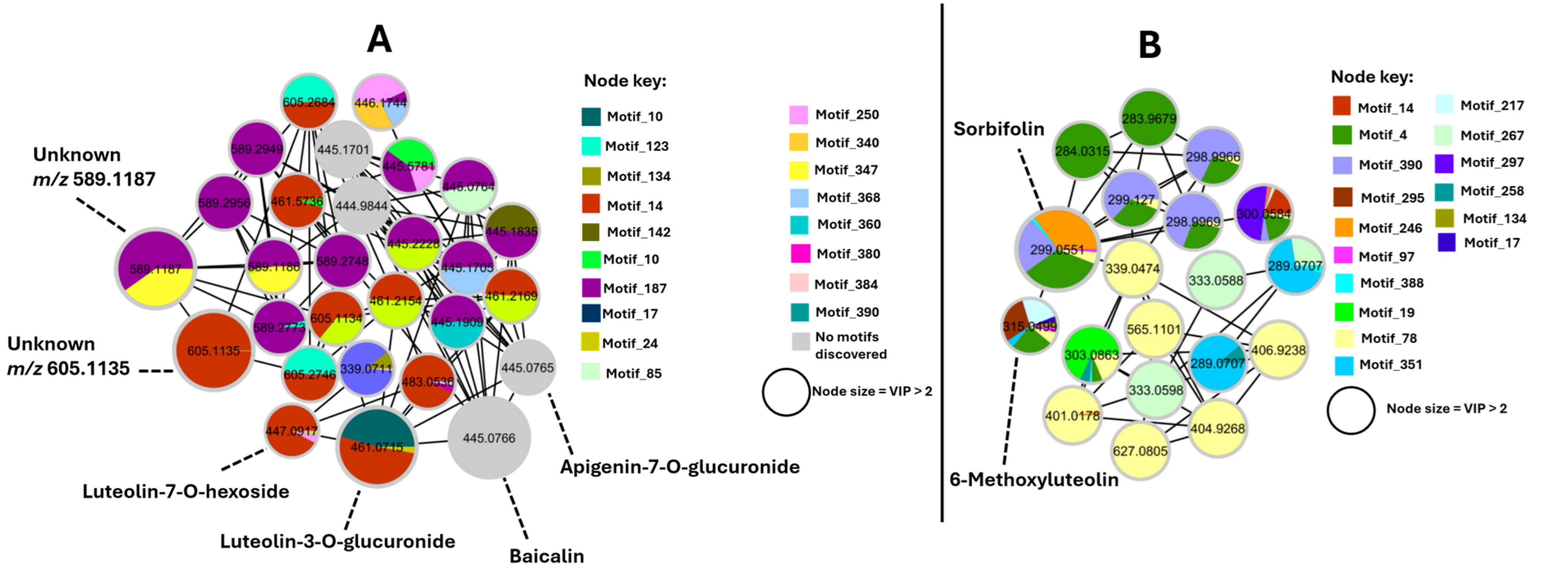

Figure S4: MS2LDA 2.0 discovered Mass2Motifs (substructures) in **Flavone** molecular families (MFs) from the generated FBMN (**Figure S1**) of *P. hadiensis* and *P. ambiguus* leaf extracts. **(A)** An MF of glycosylated flavones consisting of luteolin 3-O-glucuronide (*m/z* 605.1135), baicalin (*m/z* 445.0766), *m/z* 589.1187 and *m/z* 605.1135 as discriminating metabolite features (VIP score >2) with 18 Mass2Motifs shown as coloured pie charts on the nodes. (**B**) An MF of methylated flavones consisting of sorbifolin (*m/z* 299.0551) as a discriminating metabolite feature with 16 Mass2Motifs shown as coloured pie charts on the nodes.

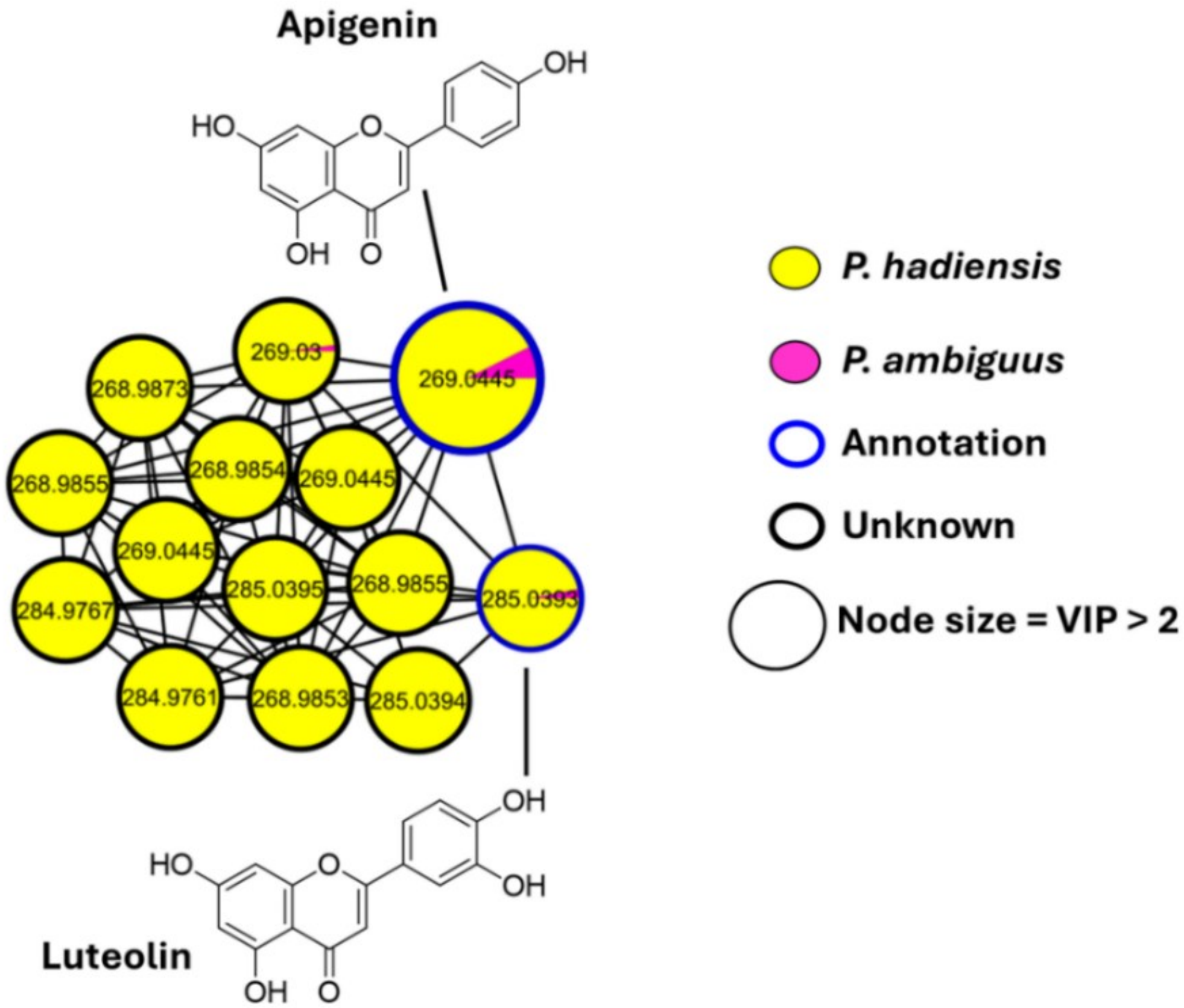

Figure S5: An MF of flavone aglycones from the generated FBMN (**Figure S1**) consisting of apigenin (*m/z* 269.0445) and luteolin (*m/z* 285.0393) as discriminating metabolite features (VIP score >2). The pie charts indicate the relative abundance of metabolite features in *P. hadiensis* and *P. ambiguus*.

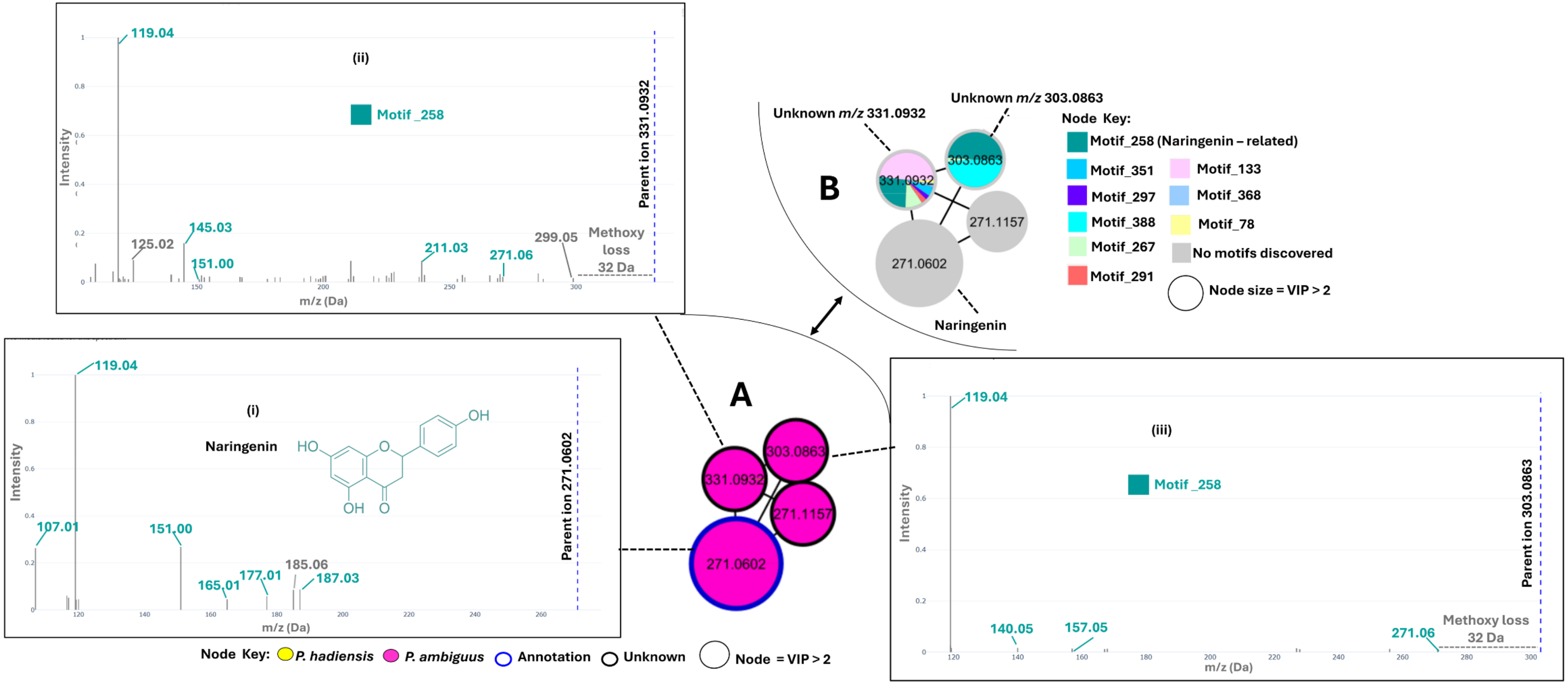

Figure S6:**MS2LDA 2.0 substructure discovery on the computed FBMN. (A)** An MF of naringenin-related metabolites with the duplicate MF (**B**) showing 9 discovered Mass2Motifs, including motif_258 (Naringenin-related) shared by unknown metabolite features, namely *m/z* 331.0932 (**ii**) and *m/z* 303.086 (**iii**). The node key at the bottom right describes MF (**A**), where the pie charts indicate the relative abundance of metabolite features across the two plants species while the pie charts in duplicate MF (**B**) indicate the discovered Mass2Motifs listed on the top right.

Table S1: All putatively annotated metabolites (level 2 and 3 MSI) in the methanolic leaf extracts of *P. ambiguus* and *P. hadiensis*. Some of the discovered key Mass2Motifs (substructures) in the putative annotations are listed in the table where some of the motifs were matched to references in the LDB_NEG_MotifDB_01, LDB_NEG_MotifDB_02 and Motifset Rhamnaceae Plant Motif Data Bases (MotifDBs) in the MS2LDA analysis.

No.	*m/z*	Rt (min)	Adduct	MF	MSI level	Fragment ions	Mass2Motif	Putative annotations	Abbrev or alternative name	Chemical class
**1**	747.5142	23.03	M-H	$C_{43}H_{76}O_{13}$	2	483, 391, 281, 255, 152	Motif_37 (255 Da) Motif_385 (281 Da)	PG 34:1	PG 34:1	Glycerophospholipid
**2**	445.0766	10.28	M-H	$C_{21}H_{18}O_{11}$	2	269, 113	Motif_187 related to Apigenin-7-O-glucuronide (445 Da, 269 Da, 175 Da, 113 Da), Motif_85 (209 Da, 183 Da)	Baicalin	Baicalin	Flavonoid
**3**	473.0713	8.69	M-H	$C_{22}H_{18}O_{12}$	2	219, 191, 179, 161, 149, 135, 112, 103	Motif_194 related to Caffeic acid (179 Da)	Chicoric acid	Ch Acid	Cinnamic acids and derivatives
**4**	359.0761	10.00	M-H	$C_{18}H_{16}O_{8}$	2	197, 179, 161, 133, 123	Motif_106 (161 Da), Motif_219 (123)	Rosmarinic acid	RA	Cinnamic acids and derivatives
**5**	721.3636	19.64	M-H	$C_{33}H_{56}O_{14}$	2	397, 277, 235, 101		Glc-Glc-octadecatrienoyl-sn-glycerol (isomer 2)	Glc-Glc	Glycerophospholipid
**6**	549.0992	9.86	M-H	$C_{24}H_{20}O_{15}$	2	505, 463, 445, 301, 300, 285, 271, 151	Motif_295 (Ref motif ID = motif_89 related to Quecetin: 301 Da, 300 Da, 271 Da, 151 Da), Motif_337 (285 Da)	Quercetin 3-O-malonylhexose	Q-3-M	Flavonoid
**7**	891.1598	10.26	[2M-H]-	$C_{21}H_{18}O_{11}$	2	445, 269, 113	Motif_187 related to Apigenin-7-O-glucuronide (445 Da, 269 Da, 175 Da, 113 Da), Motif_250 (113 Da)	Baicalein	Baicalein	Flavonoid

**8**	285.0393	11.95	M-H	$C_{15}H_{10}O_6$	2	217, 199, 175, 151, 133, 107	Motif_134 (217 Da, 217 Da, 199 Da, 175 Da, 151 Da, 133 Da, 107 Da)	Luteolin	Lut	Flavonoid
**9**	269.0445	13.01	M-H	$C_{15}H_{10}O_5$	2	159, 151, 121, 117, 107	Motif_380 (117 Da)	Apigenin	Apig	Flavonoid
**10**	315.0499	11.25	M-H	$C_{16}H_{12}O_7$	2	300, 283, 228, 227, 201, 136, 108	Motif_4 related to sorbifolin (283 Da, 227 Da, 136 Da)	6-Methoxyluteolin	6-Met-T	Flavonoid
**11**	431.0972	10.32	M-H	$C_{21}H_{20}O_{10}$	2	311, 283, 269, 117, 105	Motif_108 (311 Da, 283 Da)	Apigenin-8-C-hexose	A-8-h	Flavonoid
**12**	503.3363	15.67	M-H	$C_{30}H_{48}O_6$	2	485, 457, 453, 441, 421, 409	Motif_212 (453 Da, 441 Da, 421 Da, 409 Da)	Arjungenin	Arj	Prenol Lipid
**13**	313.0706	14.66	M-H	$C_{17}H_{14}O_6$	2	298, 283, 269, 227, 211, 185, 163	Motif_24 (Ref motif ID = motif_89 related to rhamnocitrin: 298 Da, 283 Da)	Cirsimaritin	Cirs	Flavonoid
**14**	312.1230	10.13	M-H	$C_{18}H_{19}NO$	2	190, 178, 148, 135, 107	Motif_214 (148 Da)	Feruloyltyramine	Feru	Phenols
**15**	299.0551	12.31	M-H	$C_{16}H_{12}O_6$	2	284, 283, 227, 136	Motif_4 related to sorbifolin (283 Da, 227 Da, 136 Da)	Sorbifolin	Sorb	Flavonoid
**16**	521.1280	9.46	M-H	$C_{24}H_{26}O_{13}$	2	323, 197, 179, 161, 135	Motif_362 (197 Da), Motif_106 (161 Da)	Salviaflaside	Salv	Cinnamic acids and derivatives
**17**	477.1390	9.68	M-H	$C_{23}H_{26}O_{11}$	2	323, 179, 161, 133	Motif_106 (161 Da)	Calceolarioside A	Cal A	Cinnamic acids and derivatives
**18**	719.1604	9.38	M-H	$C_{36}H_{32}O_{16}$	2	359, 197, 179, 161, 135	Motif_305 (197 Da, 179 Da, 161 Da)	Sagerinic acid	Sag Acid	Cinnamic acids and derivatives
**19**	271.0602	14.49	M-H	$C_{15}H_{12}O_5$	2	165, 151, 119, 107	Motif_258 related to naringenin (119 Da)	Naringenin	Nari	Flavonoid

**20**	447.0871	10.33	M-H	$C_{21}H_{20}O_{11}$	2	327, 284, 255, 227, 151	Motif_390 (Ref motif ID = motif_130 related to kaempferol: 284 Da, 255 Da, 227 Da)	Kaempferol-3-O-hexoside	K-3-h	Flavonoid
**21**	505.1339	9.88	M-H	$C_{23}H_{22}O_{13}$	2	445, 385, 300, 301, 285, 271, 195, 181, 165, 119	Motif_337 (285 Da)	Quercetin 3-(6"-acetylhexoside)	Q-3-acetylh	Flavonoid
**22**	463.0828	9.61	M-H	$C_{21}H_{22}O_{12}$	2	300, 271, 178, 151	Motif_295 (Ref motif ID = motif_89 related to Quecetin: 301 Da, 300 Da, 271 Da, 151 Da),	Quercetin-3-O-hexoside	Q-3-h	Flavonoid
**23**	463.0943	9.55	M-H	$C_{21}H_{22}O_{12}$	2	301, 300, 299, 271, 270, 181	Motif_295 (Ref motif ID = motif_89 related to Quecetin: 301 Da, 300 Da, 271 Da, 151 Da)	Quercetrin-3-O-galactoside	Q-3-h2	Flavonoid
**24**	311.1734	20.24	M-H	$C_{16}H_{24}O_6$	2	311, 197, 183, 149	Motif_154 (311 Da, 197 Da, 183 Da, 149 Da)	Thymol-beta-D-glucoside	Thy-D-G	Prenol lipid
**25**	445.0765	11.46	M-H	$C_{21}H_{18}O_{11}$	2	269, 175, 113	Motif_187 related to Apigenin-7-O-glucuronide (445 Da, 269 Da, 175 Da, 113 Da), Motif_85 (209 Da, 183 Da)	Apigenin-7-O-glucuronide	A-7-Glucur	Flavonoid
**26**	329.0655	13.54	M-H	$C_{17}H_{14}O_7$	2	313, 299, 285, 271, 243, 227, 199, 133		Cirsiliol	Cirsiliol	Flavonoid
**27**	447.0917	10.97	M-H	$C_{21}H_{20}O_{11}$	2	285, 151	Moitif_14 related to luteolin (285, 175, 133)	Luteolin-7-O-hexoside	L-7-glu	Flavonoid
**28**	461.0715	9.56	M-H	$C_{21}H_{18}O_{12}$	2	381, 355, 327, 285, 113	Moitif_14 related to luteolin (285, 175, 133)	Luteolin 3-O-glucuronide	L-3-glucu	Flavonoid
**29**	696.4675	24.63	M-H	$C_{39}H_{68}O_8P$	2	433, 415, 279, 152	Motif_306 (279 Da)	PA (18:2/18:2)	PA 18:2	Glycerophospholipid

**30**	177.0184	1.41	M-H	$C_9H_6O_4$	2	149, 133, 121, 105	Motif_134 (133 Da, 121 Da)	Esculetin	Escu	Coumarins and derivatives
**31**	179.0341	6.97	M-H	$C_9H_8O_4$	2	135, 117, 107, 108	Motif_200 (135 Da)	Caffeic acid	Caff Acid	Cinnamic acids and derivatives
**32**	315.1074	5.02	M-H	$C_{14}H_{20}O_8$	2	153, 135, 123	Motif_280 (153 Da), Motif_374 (123 Da)	2-Hydroxy-4-(2-hydroxyethyl)phenyl-beta-D-glucopyranoside	2-H-glucop	Organooxygen compounds
**33**	283.0965	15.59	M-H	$C_{17}H_{16}O_4$	2	179, 161, 135	Motif_106 (161 Da) Motif_200 (135 Da)	Caffeic acid phenethyl ester	Caff-acid-PE	Cinnamic acids and derivatives
**34**	283.0601	16.14	M-H	$C_{16}H_{12}O_5$	2	268, 239, 211, 117		Genkwanin	Gen	Flavonoid
**35**	285.0757	11.95	M-H	$C_{16}H_{14}O_5$	2	165, 149, 122,119	Motif_258 related to naringenin (119 Da)	Sakuranetin	Sakura	Flavonoid
**36**	293.1747	15.63	M-H	$C_{18}H_{30}O_3$	2	221, 205, 192, 177, 164	Motif_172 (221 Da, 192 Da)	13-HOTrE(r)|13S-hydroxy-6Z,9Z,11E-octadecatrienoic acid|13(S)-HOTrE(gamma)	13-HOTrE	Fatty Acyls
**37**	298.9969	12.30	M-H	$C_{16}H_{12}O_6$	2	284, 227, 211, 183, 156, 136, 117	Motif_4 related to sorbifolin (283 Da, 227 Da, 136 Da)	Hispidulin	Hispidulin	Flavonoid
**38**	549.3429	15.67	M+FA-H	$C_{36}H_{58}O_{11}$	2	485, 453, 443, 409		Olean-12-en-28-oic acid, 2,3,19,23-tetrahydroxy-, 2(2alpha,3beta,5xi,9xi,19alpha)-	Olean-12	Prenol Lipid
**39**	571.2876	22.19	M-H	$C_{25}H_{48}O_{12}$	2	391, 315, 255, 241, 152	Motif_37 (255), Motif_52 (241 Da), Motif_180 (152)	PI (16:0/0:0)	PI 16:0	Glycerophospholipid
**40**	595.2876	21.60	M-H	$C_{27}H_{48}O_{12}$	2	415, 315, 279, 241, 152	Motif_306 (279 Da)	PI (18:2/0:0)	PI 18:2	Glycerophospholipid

**41**	721.4999	22.15	M-H	$C_{38}H_{75}O_{10}$	2	483, 391, 255, 171, 152	Motif_144 (255 Da)	PG 32:0	PG 32:0	Glycerophospholipid
**42**	359.098	6.20	M-H	$C_{15}H_{20}O_{10}$	2	197, 182, 167, 153, 138, 123		Benzoic acid + 1O, 2MeO, O-Hex	BA+10	Tannins
**43**	327.2166	14.31	M-H	$C_{18}H_{32}O_5$	2	211, 183, 171, 137	Motif_215 (183 da, 171 Da, 137 Da)	FA 18:2+3O	FA 18:2+3O	Fatty Acyls
**44**	431.1911	10.27	M-H	$C_{21}H_{20}O_{10}$	2	269, 268, 151		Apigenin-7-O-hexoside	A-7-h	Flavonoid
**45**	431.0972	10.32	M-H	$C_{21}H_{20}O_{10}$	2	311, 269, 268, 239		Apigenin-4'-hexoside	A-4-h	Flavonoid
**46**	283.0601	16.14	M-H	$C_{16}H_{12}O_5$	2	268, 239, 211, 171, 151, 107		Methyl-4'-Apigenin	Me-4-Apig	Flavonoid
**47**	697.2331	11.34	M+FA-H	$C_{31}H_{40}O_{15}$	2	651, 475, 193, 175, 160, 113	Motif_135 (175 Da)	[5-hydroxy-6-[2-(4-hydroxy-3-methoxyphenyl)ethoxy]-2-(hydroxymethyl)-4-(3,4,5-trihydroxy-6-methyloxan-2-yl)oxyoxan-3-yl] (E)-3-(4-hydroxy-3-methoxyphenyl)prop-2-enoate	5-hydr-enoate	Cinnamic acids and derivatives
**48**	487.3415	18.50	M-H	$C_{30}H_{48}O_5$	2	469, 423, 407	Motif_349 (469 Da)	Arjunic acid	Arju Acid	Prenol Lipid
**49**	721.5014	21.27	M-H	$C_{38}H_{75}O_{10}$	2	483, 465, 391, 255, 171, 152	Motif_144 (255 Da)	[3-[[2,3-dihydroxypropoxy]-hydroxyphosphoryl]oxy-2-hexadecanoyloxy	2,3-dihy-anoate	Glycerophospholipid

								propyl] hexadecanoate		
**50**	261.1122	14.81	M-H	$C_{15}H_{18}O_4$	2	217, 189, 173, 157,	Motif_332 (217 Da, 189 Da, 173 Da)	(4aR,9aR)-8,8-dimethyl-1-methylidene-2-oxo-4a,6a,7,9-tetrahydro-4H-pentaleno[1,6a-c]pyran-5-carboxylic acid	Dime-car-acid	Prenol Lipid
**51**	431.0972	10.32	M-H	$C_{21}H_{20}O_{10}$	2	268, 240, 152		Sophoricoside	Sophor	Flavonoid
**52**	447.0921	9.61	M-H	$C_{21}H_{20}O_{11}$	2	327, 285, 271, 151, 107	Motif_281 (151 Da, 125 Da, 107 Da)	Kaempferol-3-O-hexoside	Kaemp-3-h2	Flavonoid
**53**	339.0711	6.24	M-H	$C_{15}H_{16}O_9$	2	177, 149, 133, 120, 105	Motif_290 (117 Da)	6,7-Dihydroxycoumarin-6-glucoside	6,7-Dihy-6-g	Coumarins and derivatives
**54**	329.0655	13.54	M-H	$C_{17}H_{14}O_7$	2	314, 299, 271, 243, 227, 199		Cirsiliol	Cirsiliol	Flavonoid
**55**	579.2071	10.07	M-H	$C_{28}H_{36}O_{13}$	2	417, 402, 387, 371		Acanthoside B	Acan B	Lignans
**56**	461.1651	8.73	M+FA-H	$C_{21}H_{15}O_{12}$	2	191, 149, 131, 113, 101	Motif_298 (149 Da, 131 Da, 113 Da, 101 Da)	Beta-D-Glucopyranoside, 2-phenylethyl 6-O-beta-D-xylopyranosyl	B-D-Xylop	Organooxygen compounds
**57**	559.3110	20.63	[M+FA]-	$C_{27}H_{46}O_9$	2	277, 253, 157	Motif_35 (227 Da)	9,12,15-Octadecatrienoic acid, 3-(hexopyranosyloxy)-2-hydroxypropyl	Octa-2-hexopy	Glycerophospholipids

								ester, (9Z,12Z,15Z)		
**58**	463.0870	8.85	M-H	$C_{21}H_{20}O_{12}$	2	287, 161, 151, 135, 125 113, 107	Motif_281 (151 Da, 125, 107 Da)	(2S)-eriodictoyl-7-O-beta-D-glucopyranosidur onic acid	Erio-7-O-glucur	Flavonoid
**59**	433.1129	8.96	M-H	$C_{21}H_{22}O_{10}$	2	179, 161, 135, 133, 113, 101	Motif_106 (161 Da)	6-O-Caffeoylarbutin	6-O-Caff	Cinnamic acids and derivatives
**60**	593.1655	7.68	M-H	$C_{27}H_{30}O_{15}$	2	575, 545, 503, 485, 473, 353, 311		Apigenin 6,8-dihexoside	A-6,8-dihex	Flavonoid
**61**	593.1499	8.35	M-H	$C_{27}H_{30}O_{15}$	2	473, 383, 353, 311		Vicenin 2	Vicenin 2	Flavonoid
**62**	467.2118	11.91	[M+FA]-	$C_{19}H_{34}O_{15}$	2	289, 161, 113, 101	Motif_228 (113 Da, 101 Da)	1-Octen-3-yl primeveroside	1-Octen-Prim	Fatty Acyls
**63**	193.0498	9.82	M-H	$C_{10}H_{10}O_4$	2	161, 133	Motif_106 (161 Da)	Ferulic acid	Ferulic acid	Cinnamic acids and derivatives
**64**	717.1448	9.50	M-H	$C_{36}H_{30}O_{16}$	2	537, 519, 493, 339, 295, 179	Motif_160 (519 Da, 339 Da, 295 Da)	(2R)-2-[(E)-3-[3-[(1R)-1-carboxy-2-(3,4-dihydroxyphenyl)ethoxy]carbonyl-2-(3,4-dihydroxyphenyl)-7-hydroxy-2,3-dihydro-1-benzofuran-4-yl]prop-2-enoyl]oxy-3-(3,4-dihydroxyphenyl)propanoic acid	SCHEMBL14911156	Lignans
**65**	293.1747	15.63	M-H	$C_{17}H_{26}O_4$	2	236, 221, 192, 177	Motif_172 (221 Da, 192 Da)	6-Gingerol	6-Gingerol	Phenol

**66**	711.3946	12.31	M+FA-H	$C_{38}H_{58}O_{11}$	2	665, 503, 207		[(2alpha,3beta,5xi,6beta,9xi,18xi)-2,3,6,23-Tetrahydroxy-28-oxoolean-12-en-28-yl]-beta-D-glucopyranose	NCGC00180568-03	Prenol Lipid
**67**	327.2166	14.59	M-H	$C_{18}H_{32}O_5$	2	291, 239, 229, 221, 211, 193, 183, 171, 127	Motif_215 (171 Da, 127 Da)	(10E,15E)-9,12,13-trihydroxyoctadeca-10,15-dienoic acid	Trihy-dien-A	Fatty Acyls
**68**	357.0605	8.05	M-H	$C_{18}H_{14}8$	2	269, 254, 237, 159, 147, 135, 131, 109	Motif_17 (159 Da, 135 Da, 109 Da)	2,3-Dihydro-2-(3,4-dihydroxyphenyl)-3-carboxy-1,4-benzodioxin-6-acrylic acid	DDBA	Benzodioxanes
**69**	363.1797	18.64	M-H	$C_{20}H_{28}O_6$	2	301, 283	Motif_1 (301 Da)	Rabdoternin A	Rabdoternin A	Prenol lipid
**70**	497.2739	14.11	M-H	$C_{26}H_{42}O_9$	2	335, 317		(5β, 8α, 9β, 11β, 13α)-11, 13,15-Trihydroxykaur-16-en-3-yl-beta-D-allopyranoside	Trihydroxykaur	Prenol lipid
**71**	250.0711	7.43	M-H	$C_{12}H_{13}NO$	2	132, 113, 114		Phenylacetylaspartic acid	PAA	Amino acid derivatives
**72**	290.0869	1.31	M-H	$C_{11}H_{17}NO$	2	200, 155, 128		N-Fructosyl pyroglutamate	NFP	Amino acid derivatives
**73**	137.0235	5.17	M-H	$C_7H_6O_3$	2	137, 136, 119, 109, 108		Protocatechuic aldehyde	PCA	Phenols
**74**	341.0867	6.83	M-H	$C_{15}H_{18}O_9$	2	179, 177, 161, 151, 149, 135	Motif_200 (135 Da)	Caffeic acid hexoside	CAH	Cinnamic acids and derivatives

**75**	153.0184	3.37	M-H	$C_7H_6O_4$	2	109, 108	Motif_170 (153 Da, !09 Da, 108 Da)	2,5-dihydroxybenzoic acid	2,5-dihydro	Benzodioxanes
**76**	167.0341	3.03	M-H	$C_8H_8O_4$	2	123, 122, 108	Motif_170 (153 Da, !09 Da, 108 Da)	Homogentisic acid	HA	Benzodioxanes
**77**	361.1647	19.29	M-H	$C_{20}H_{26}O_6$	2	346, 179, 165		2,3-bis[(4-hydroxy-3-methoxyphenyl)methyl]butane-1,4-diol	2,3-Bis	Lignans
**78**	328.1179	8.75	M-H	$C_{18}H_{19}NO$	2	161, 148, 134, 133, 135	Motif_106 (161 Da), Motif_381 (135 Da)	N-trans-Feruloyloctopamine	NTF	Cinnamic acids and derivatives
**79**	295.2267	19.78	M-H	$C_{18}H_{32}O_3$	2	277, 195, 171, 125	Motif_215 (171 Da)	9-hydroxy-10,12-octadecadienoic acid	9-hydroxy	Fatty Acyls
**80**	431.0972	10.32	M-H	$C_{21}H_{20}O_{10}$	2	311, 269, 240		Emodin-8-Beta-D-Glucoside	E-8-BD-G	Phenols
**81**	305.1746	16.11	M-H	$C_{18}H_{32}O_3$	2	261, 191	Motif_59 (191 Da, 123 Da)	Amorfrutin 2	Amorfrutin 2	Benzodioxanes
**82**	721.5008	22.73	M-H	$C_{38}H_{75}O_{10}$	2	255	Motif_144 (255 Da)	PG (16:0/16:0)	PG (16:0/16:0)	Glycerophospholipids
**83**	311.2215	17.52	M-H	$C_{38}H_{75}O_{10}$	2	293, 183	Motif_154 (183 Da)	9-Hydroperoxy-11,12-octadecadienoic acid	9-HPODE	Fatty Acyls
**84**	297.0394	11.87	M-H	$C_{16}H_{10}O_6$	2	254, 226, 198		Fallacinal	Fallacinal	Phenols
**85**	435.2505	23.10	M-H	$C_{21}H_{41}O_7$	2	153		1-Oleoyl-L-.alpha.-lysophosphatidic acid	1-Oleo	Glycerophospholipids
**86**	501.3572	22.73	[M+HCOO]-	$C_{30}H_{48}O_3$	2	455		Betulinic acid	Betulinic acid	Prenol Lipids

**87**	301.0622	12.29	M-H	$C_{16}H_{14}O_6$	2	286, 151, 119	Motif_291 (286 Da)	3',5,7-Trihydroxy-4'-methoxyflavanone	4-Tri-methoxy	Flavonoids
**88**	433.1327	9.65	M-H	$C_{16}H_{14}O_6$	2	271, 151, 119	Motif_258 related to naringenin (119 Da)	naringenin-7-O-hexoside	Nar-hexo	Flavonoids
**89**	577.1338	13.52	M-H	$C_{27}H_{30}O_{14}$	2	431, 269, 268, 145	Motif_187 related to Apigenin-7-O-glucuronide (445 Da, 269 Da, 175 Da, 113 Da), Motif_85 (209 Da, 183 Da)	Apigenin-7-O-neohesperidoside	Api-neoh	Flavonoids
**90**	693.2385	11.66	M-H	$C_{32}H_{38}O_{17}$	2	193, 175, 160	Motif_135 (175 Da)	Helonioside A	Helonioside A	Cinnamic acids and derivatives
**91**	331.1905	20.34	M-H	$C_{20}H_{28}O_4$	2	313, 303, 299, 287, 267, 193, 165, 129, 122		7|A,15-Dihydroxydehydroabietic acid	DDA	Prenol Lipids
**92**	144.0446	8.82	M-H	C_9H_7NO	2	126, 116, 115,		Indole-3-carboxyaldehyde	I-3-C	Amino acid derivatives
**93**	303.0500	5.28			3	211, 161, 119, 117	Motif_258 related to naringenin (119 Da)	Flavonoid/flavone or flavonol		Flavonoids
**94**	299.2006	19.37			3	283, 269, 227, 144, 126		Abietane diterpenoid related to dehydroabietic acid		Prenol Lipids
**95**	593.1113	7.09			3	307, 285, 153, 108	Motif_170 (153 Da, 108 Da)	Glycosylated flavanol		Flavonoids
**96**	373.0917	10.55			2	285, 267, 255		Protocetraric acid		
**97**	609.1446	8.22	M-H	$C_{27}H_{30}O_{16}$	2	447, 327, 285	Motif_110 (447 Da 327 Da), Moitif_14 related to luteolin (285, 175, 133)			Flavonoids
**98**	593.1287	12.31			3	323, 269, 221, 161				Flavonoids

**99**	605.1135	10.71			3	543, 503, 461, 443, 285	Moitif_14 related to luteolin (285, 175, 133)			Flavonoids
**100**	589.1187	11.46			3	445, 427, 269, 113	Motif_187 related to Apigenin-7-O-glucuronide (445 Da, 269 Da, 175 Da, 113 Da), Motif_85 (209 Da, 183 Da)			Flavonoids
**101**	303.0863	13.91			3	119	Motif_258 related to naringenin (119 Da)			Flavonoids
**102**	331.0932	11.54			3	299, 271, 200, 145, 119	Motif_258 related to naringenin (119 Da)			Flavonoids
